# The Effects of Internet-Based Storytelling Programs (Amazing Adventure Against Stigma) in Reducing Mental Illness Stigma With Mediation by Interactivity and Stigma Content: Randomized Controlled Trial

**DOI:** 10.2196/37973

**Published:** 2022-08-12

**Authors:** Tiffany H C Fong, Winnie W S Mak

**Affiliations:** 1 Department of Psychology The Chinese University of Hong Kong Hong Kong Hong Kong

**Keywords:** mental illness stigma, internet-based, interactivity, storytelling, social distance, microaggression

## Abstract

**Background:**

Mental illness stigma has been a global concern, owing to its adverse effects on the recovery of people with mental illness, and may delay help-seeking for mental health because of the concern of being stigmatized. With technological advancement, internet-based interventions for the reduction of mental illness stigma have been developed, and these effects have been promising.

**Objective:**

This study aimed to examine the differential effects of internet-based storytelling programs, which varied in the levels of interactivity and stigma content, in reducing mental illness stigma.

**Methods:**

Using an experimental design, this study compared the effects of 4 storytelling websites that varied in the levels of interactivity and stigma content. Specifically, the conditions included an interactive website with stigma-related content (*combo* condition), a noninteractive website with stigma-related content (*stigma* condition), an interactive website without stigma-related content (*interact* condition), and a noninteractive website without stigma-related content (*control* condition). Participants were recruited via mass emails to all students and staff of a public university and via social networking sites. Eligible participants were randomized into the following four conditions: *combo* (n=67), *stigma* (n=65), *interact* (n=64), or *control* (n=67). The participants of each group viewed the respective web pages at their own pace. Public stigma, microaggression, and social distance were measured on the web before the experiment, after the experiment, and at the 1-week follow-up. Perceived autonomy and immersiveness, as mediators, were assessed after the experiment.

**Results:**

Both the *combo* (n=66) and *stigma* (n=65) conditions were effective in reducing public stigma and microaggression toward people with mental illness after the experiment and at the 1-week follow-up. However, none of the conditions had significant time×condition effects in reducing the social distance from people with mental illness. The *interact* condition (n=64) significantly reduced public stigma after the experiment (*P*=.02) but not at the 1-week follow-up (*P*=.22). The *control* condition (n=67) did not significantly reduce all outcomes associated with mental illness stigma. Perceived autonomy was found to mediate the effect of public stigma (*P*=.56), and immersiveness mediated the effect of microaggression (*P*=.99).

**Conclusions:**

Internet-based storytelling programs with stigma-related content and interactivity elicited the largest effects in stigma reduction, including reductions in public stigma and microaggression, although only its difference with internet-based storytelling programs with stigma-related content was not statistically significant. In other words, although interactivity could strengthen the stigma reduction effect, stigma-related content was more critical than interactivity in reducing stigma. Future stigma reduction efforts should prioritize the production of effective stigma content on their web pages, followed by considering the value of incorporating interactivity in future internet-based storytelling programs.

**Trial Registration:**

ClinicalTrials.gov NCT05333848; https://clinicaltrials.gov/ct2/show/NCT05333848

## Introduction

### Mental Illness Stigma

According to the social cognitive model of stigma, mental illness stigma is defined as having stereotypical thoughts, prejudicial feelings, and discriminatory actions against people with mental illness in situations with power differentials [1]. Mental illness stigma is a global concern owing to its detrimental effects imposed on people with mental illness across various life domains (eg, education, housing, employment, and health care) during their recovery and their willingness to seek help [1-3].

Given the possible negative impacts of mental illness stigma, various approaches have been proposed to reduce it, with education and contact being identified as the 2 most common and effective approaches in inducing positive attitudinal changes and reducing public stigma [4-7]. Knowledge enhancement through psychoeducation can improve mental health literacy, which corrects misunderstanding of mental health–related issues, challenges stigmatizing beliefs, and supports recovery from mental illness [7]. Contact is effective in confronting stigmatization through an equal, interpersonal exchange that fosters perspective taking and empathy [4]. The primary form of contact is an in vivo one, which requires the person with lived experience to share his or her experiences of mental illness and stigmatization with an audience live. However, it can be costly and taxing for speakers to share their experiences repeatedly [8]. Hence, researchers have investigated the possibilities of using parasocial contacts, which include video-based, filmed, and web-based contacts [4,8-11]. These have exhibited similar effects as were found with in vivo contacts [4,11].

### Internet-Based Stigma Reduction Interventions

Recently, internet-based programs addressing mental illness stigma have been established worldwide owing to their low cost, accessibility, and scalability [12-14]. Studies have shown the effectiveness of internet-based stigma reduction programs in the form of social media platforms, digital games, webinars, filmed social contact, and websites [9,15-19]. Research has also shown that internet-based and face-to-face stigma reduction programs are equally effective [20,21]. Research has also found increased empathy and reduced intergroup anxiety to be significant mediators explaining the effects of interventions on the reduction of mental illness stigma [22,23]. However, the content and design of these internet-based stigma reduction programs vary greatly, and limited efforts have been made to investigate the common factors contributing to their effectiveness.

In addition to incorporating the critical determinants, namely education and contact, in stigma reduction, many internet-based interventions have made use of interactivity and storytelling in their designs and have demonstrated positive results in reducing mental illness stigma [24-28]. However, the types of interactivity are diverse, and it is unknown whether the addition of interactivity induces significant positive attitudinal changes that should be valued. Despite the popularity of incorporating elements of interactivity into websites to enhance the engagement of users, there is minimal empirical work investigating the effect of interactivity on stigma reduction. Thus, rather than focusing on psychological variables (ie, empathy and intergroup anxiety) as mediators (the effects of which have been established), this study focused on the mediators related to the form of interventions and aimed to examine the differential effects of internet-based storytelling programs with the presence or absence of interactivity and stigma content in reducing mental illness stigma.

### Stigma-Related Content

As previously mentioned, educating about mental illness and fostering an understanding toward people with mental illnesses helps individuals to clear misunderstandings and empathize with their experiences [7]. Hence, it is pivotal to introduce stigma-related content into stigma reduction programs. However, research has shown significant positive attitudinal changes in *control* conditions where stigma-related content is not present [28,29]. Although the situation is rare, it is proposed that the effect might be attributed to the social desirability effect and priming effect in which people have been prompted to answer questions related to mental illness attitudes at preassessment [28,29]. In this study, stigma-related content was one of the independent variables that accounted for this possible effect. Despite some cases of stigma reduction in the absence of stigma content, we hypothesized that the reduction of stigma will only manifest in conditions with the presence of stigma content.

### Storytelling

In previous research related to interventions aimed at reducing mental illness stigma, the use of storytelling has demonstrated significant reduction in stigma [24,28,30]. Corrigan and Kosyluk [31,32] have identified three crucial components that make storytelling effective in reducing mental illness stigma, namely people with lived experience as storytellers, in-person story delivery, and descriptions encompassing both ups and downs on the recovery journey of mental illness. Disclosure from storytellers enables people to understand the experience shared in a deeper way with the aid of contexts and connection with the storytellers [33]. Storytelling was found to be positively related to people’s reflection, motivation, and engagement, which not only allows people to have a better understanding of people with mental illnesses cognitively but also cultivates empathy in people [30].

### Interactivity

Empirically, few studies have shown that communication using interactive media could exert synergistic effects with education in reducing stigmatizing attitudes toward people with mental illness [26,27,34]. However, past efforts investigating internet-based stigma reduction programs that have incorporated interactivity have mainly used interactivity in different delivery formats. For instance, a study allowed participants to choose the sequence of reading materials but not the content [26]. Another study allowed individuals to undergo simulated contact by using web-based interactions with avatars who shared their emotional distress [27]. There is still a dearth of empirical studies investigating the effects of interactivity in internet-based stigma reduction programs.

Scholars have proposed that the effect might be attributed to the inducement of positive affect during the processing of mental illness information on the internet [26,35]. According to the Systemic Thinking Model, in interactive environments, interactivity allows individuals to be the agent and effect physical environmental changes that best align with their thinking needs and flow [36]. Individuals actively manipulate information and receive contingent feedback, which facilitates the encoding of new information [37]. In turn, information processing and learning are facilitated [36]. Empirical research has found that interactivity induces positive affect, including increased satisfaction and pleasure [38]. Individuals tend to favor interactive information through attitude transfer mechanisms [39]. In addition, research shows that incidental positive affect can reduce complex affective judgments toward outgroup members and lead to more prosocial orientations [40]. These findings suggest that interactivity could lead to positive affect and reduced stigmatizing attitudes toward people with mental illness even without the presence of stigma content aiming to correct misconceptions.

Interactivity is a favorable element in learning. McMillan proposed four main types of interaction, namely user-to-user, user-to-content, user-to-medium, and medium-to-medium interactions [41]. Most internet-based programs have used user-to-content interaction, where individuals can interact with information on the internet [42]. Kim and Stout [26] examined a type of user-to-content interaction by allowing individuals to control the navigation sequence of web pages. In this study, individuals interacted with the contents of web pages by choosing their actions and responses, and the possible factors mediating the interactivity effect were analyzed.

### Possible Mechanisms of Change

Research has found interactivity to have a significant role in improving information processing through enhanced motivation, which facilitates stigma reduction [26]. Perceived autonomy and immersiveness have been found to enhance motivation [43,44]. Thus, they might be possible mediators of the effect of interactivity although their relationships remain untapped.

Perceived autonomy refers to the perception of being the agent, which makes one consider the experience and behavior as concordant with one’s integrated sense of the self [45]. When perceived autonomy is high, autonomy need satisfaction is achieved and cognitive changes are facilitated [46]. By allowing individuals to select their responses as an avatar on the website, the heightened perceived autonomy might facilitate the endorsement of responses and actions selected, which may foster cognitive changes. Therefore, perceived autonomy might be a possible mediator of cognitive changes that correct misbeliefs about mental illnesses.

Immersiveness indicates the subjective feelings of participating in a comprehensive and realistic experience [47]. Storytelling has been evident in inducing immersiveness [48]. With higher levels of immersiveness, individuals are driven to take in messages conveyed in the story [49], which in turn leads to cognitive and affective changes related to mental illness stigma [50-53]. Interactivity also allows people to engage with the content, which is also considered an immersive medium [47]. Thus, immersiveness was proposed to be another possible mediator leading to stigma reduction.

### Aims and Hypotheses

This experimental study aimed to investigate the effect of internet-based storytelling programs on the manipulation of stigma-related content and interactivity. We hypothesized that an internet-based storytelling program with a combination of interactivity and stigma content would lead to the most significant reduction in public stigma, microaggression, and social distance from people with mental illnesses, followed by an internet-based storytelling program with stigma content only and interactivity only, compared with the control group. Second, we hypothesized that the effects observed in stigma reduction would be mediated by perceived autonomy and immersiveness owing to the presence of interactivity.

## Methods

### Study Design

This experimental study compared the following four internet-based storytelling programs: an interactive stigma content website (*combo* condition), a noninteractive stigma content website (*stigma* condition), an interactive nonstigma content website (*interact* condition), and a noninteractive nonstigma content website (*control* condition).

### Ethics Approval

Ethics approval for behavioral research was obtained from the Survey and Behavioral Research Ethics Committee of the Chinese University of Hong Kong, and the study was registered on ClinicalTrials. gov (NCT05333848). The findings of this study were reported in accordance with the recommendations of the CONSORT (Consolidated Standards of Reporting Trials) guidelines.

### Participants

This study targeted people who were aged ≥18 years and able to read and understand Chinese. Recruitment was performed by sending mass emails to students and staff of a public university in Hong Kong and by posting advertisements on social media. Individuals who were interested in participating in the study visited the registration link, where they were screened through a web-based survey on basic contact information and age. The experimenter (THCF) then provided eligible individuals a Zoom (Zoom Video Communications Inc) appointment link, where individuals indicated their preferred day and time to participate in the experiment. A Zoom link was provided to individuals upon the completion of their booking. At the scheduled Zoom experimental session, participants were given detailed information about the study aims, length of the program, and participant involvement. Participants provided informed consent by checking the *I agree* button at the end of the study description page. Afterward, participants received another web-based questionnaire link to complete the pre-experiment questionnaire. The participants were randomly assigned to 1 of the 4 experimental conditions through block randomization. Participants completed the pre-experiment, postexperiment, and 1-week follow-up questionnaires on the web.

### Storytelling Programs

The four internet-based storytelling programs were administered via the internet with 4 different web pages that were displayed in the Chinese language. The design of the web pages, including the story content and the use of graphics, was created to appeal widely to the adult population without catering specifically to a certain gender or age group. The Amazing Adventure Against Stigma website [54] was used in the experimental study for the *combo* and *stigma* conditions. Animation with fictional backgrounds (eg, mountains, canyons, and safari) and avatars of diverse body types, height, skin color, and gender-neutral hairstyle and clothing were used to minimize the effect of cultural and environmental effects that may be more prominent in using real persons and real settings on the participants and to maximize the possibility of adults with diverse backgrounds relating to the avatars. It is also easier and more economical to create control animations by using existing software than to produce videos with real people in real-life settings. Each web page took approximately 20 minutes to browse through. The presence or absence of interactivity and the presence or absence of stigma content were manipulated on the four web pages. All web pages involved a story. For the *combo* and *stigma* conditions, the story was identical, which was about the journey of a person experiencing mental illness stigma. For the *interact* and *control* conditions, the story was also identical and nonstigma related, illustrating the typical day of a person. Interactivity was manipulated by adding interactive elements to the *combo* and *interact* conditions, where participants could choose their actions and responses on web pages. In these 2 conditions, the participants had internet-based contact with the protagonist in the story, where they learned about the life experiences of the protagonist through the story portrayed on the web pages. Contact and interaction with the protagonist were in the moment as the story regarding the lived experience of the protagonist unfolded along the journey and the participants chose their responses to continue their interaction with the protagonist. The story content in the *combo* and *stigma* conditions was organized based on the disclosure of a person with a lived experience of mental illness. The person with a lived experience of mental illness accompanied participants to visualize their microaggressive encounters in various life domains (eg, work, family, and social circle) and the public’s misunderstanding of mental illness with the aid of visual images on the web page. The story also incorporated messages about the interconnection between people with or without mental illness. The selection of the aforementioned story content was based on the previous identification of both education and contact as the most effective approaches for inducing stigma reduction [4-7].

The story content in the *interact* and *control* conditions formulated a typical day for a person, which began with the morning routine, followed by having breakfast, going to work, working encounters, and ending the day. The ordinary storyline was created to minimize affective arousal and, in turn, minimize influences on judgment and decision-making according to the affect-as-information framework proposed by Storbeck and Clore [55]. At the end of each web page experience, participants were provided with a questionnaire link that measured microaggression, public stigma, social distance from people with mental illness, perceived autonomy, and immersiveness. One week after the experimental session, participants completed a follow-up questionnaire assessing microaggression, public stigma, and social distance from people with mental illness. Finally, participants were debriefed. Participants could not reaccess the contents of the web pages after the experimental session. Screenshots of the web page interventions are provided in Multimedia Appendix 1.

### Measures

#### Baseline Measures

At baseline, participants provided demographic and background information, including age, gender, education level, religion, and previous experience with mental illness.

#### Contact With People Having Mental Illness

To assess one’s previous experience with mental illness, the Level of Contact Report [56] was used, where participants indicated whether they had the experiences reported in the 12 items such as “I have watched a movie or television show in which a character depicted a person with mental illness” and “I have observed persons with a severe mental illness on a frequent basis.” Higher scores indicated higher levels of previous contact with people with mental illnesses.

#### Mental Illness Stigma Measures

##### Public Stigma Toward People With Mental Illness

The 21-item Public Stigma Scale-Mental Illness-Short Version [57] was used to assess public stigma regarding mental illness and personal advocacy. Each item was rated on a 6-point Likert scale ranging from 1 (strongly disagree) to 6 (strongly agree). Sample items included “People with mental illness are a burden to society” (public stigma) and “I wholeheartedly fight for the rights of people with mental illness” (personal advocacy). Reverse scoring was performed for personal advocacy items. Higher scores indicate higher levels of public stigma. In this study, its Cronbach α values were .93, .95, and .94 at baseline, after the experiment, and at the 1-week follow-up, respectively.

##### Microaggression

Microaggression was measured using the 17-item Mental Illness Microaggressions Scale [58], which covers the assumption of inferiority, patronization, and fear of mental illness. Each item was rated on a 4-point Likert scale ranging from 1 (strongly disagree) to 4 (strongly agree). Sample items included “If someone I’m close to told me that they had a mental illness diagnosis, I would expect them to have trouble understanding some things” (assumption of inferiority), “If someone I’m close to told me that they had a mental illness diagnosis, I would give them advice on how to remain stable” (patronization), and “If I saw a person who I thought had a mental illness in public, I would keep my distance from them” (fear of mental illness). Higher scores indicate higher levels of microaggression. In this study, the Cronbach α values of the Mental Illness Microaggressions Scale were .78, .86, and .87 at baseline, after the experiment, and at the 1-week follow-up, respectively.

##### Social Distance From People With Mental Illness

The 8-item Social Distancing Scale [57] was used to measure the behavioral intention to maintain social distance from people with mental illness. Participants rated the extent to which they endorsed each item from 1 (very willing) to 6 (very unwilling) on items such as “Assuming you have children, you will let persons with mental illnesses take care of your children” and “You will work with persons with mental illnesses in the same institution.” In this study, its Cronbach α values were .83, .88, and .86, at baseline, after the experiment, and at the 1-week follow-up, respectively.

#### Mediators Measures

##### Perceived Autonomy

To assess the perceived autonomy of the web page experience, the 10-item Self-Determination Scale [59] was used in the postexperiment questionnaire. Each item was a pair of opposite statements, in which participants rated their level of perceived choice and self-awareness with a slider from 1 (only A feels true) to 5 (only B feels true). Sample items included item—“A. During this web page experience, I always feel like I choose the things I do. B. During this web page experience, I sometimes feel that it’s not really me choosing the things I do” (perceived choice)—and item 2—“A. During this web page experience, my emotions sometimes seem alien to me. B. During this web page experience, my emotions always seem to belong to me” (self-awareness). Reverse scoring was performed for perceived choice items. In this study, its Cronbach α was .89 after the experiment.

##### Immersiveness

The 15-item Transportation Scale [60] was used to assess participants’ immersiveness in the web experience. It used a 4-point Likert scale ranging from 1 (very much) to 4 (not at all) for items such as “I could picture myself in the scene of the events described in the web page.” The last 4 items were adapted to suit the experimental conditions. In the *combo* and *stigma* conditions, the last four items were “While reading the web page, I had a vivid image of the avatar representing me”; “While reading the web page, I had a vivid image of the host”; “While reading the web page, I had a vivid image of the journey”; and “While reading the web page, I had a vivid image of the dialogue.” In the *interact* and *control* conditions, the last four items were “While reading the web page, I had a vivid image of the avatar representing me”; “While reading the web page, I had a vivid image of my home”; “While reading the web page, I had a vivid image of my breakfast”; and “While reading the web page, I had a vivid image of my office.” Items 2, 5, and 9 were framed negatively. All the items were scored in the direction that higher scores indicate higher levels of immersiveness. In this study, its Cronbach α was .84 after the experiment.

### Data Analysis

All analyses were conducted using SPSS (version 27.0; IBM Corporation) and the moderation and mediation plug-in PROCESS. Categorical chi-square and 1-way ANOVA were used to examine baseline differences among the experimental conditions. Repeated measures ANOVA with Bonferroni adjustment and post hoc analysis were conducted to detect significant interaction effects between condition and time to see if conditions showed significant reduction in all mental illness stigma outcomes across the 3 time points. Mediation analysis was conducted using PROCESS model 4 to investigate the relationship of possible mediators, perceived autonomy, and immersiveness, with all outcomes at follow-up assessment.

## Results

### Participant Characteristics

**Figure 1:**
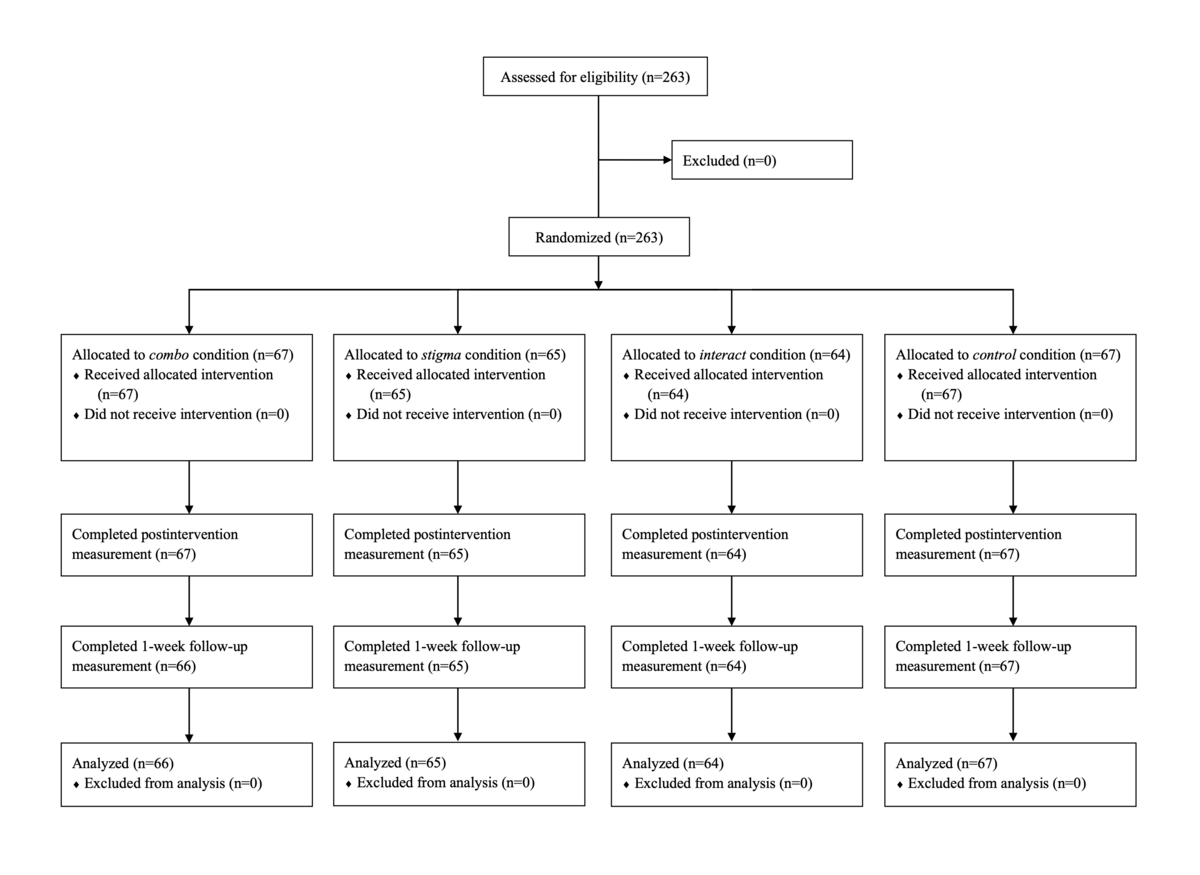
CONSORT (Consolidated Standards of Reporting Trials) diagram of participant recruitment. *Combo* condition: interactivity present; stigma content present. *Control* condition: interactivity absent; stigma content absent. *Interact* condition: interactivity present; stigma content absent. *Stigma* condition: interactivity absent; stigma content present.

A total of 263 participants were recruited for this study and completed the experimental session, pre-experiment and postexperiment questionnaires. All but 1 participant (262/263, 99.6%) completed the 1-week follow-up questionnaire. The procedure of the study is illustrated in Figure 1. Demographics and baseline characteristics of 263 participants were analyzed, and data from 262 participants were analyzed using repeated measures ANOVA and mediation analyses. The mean age of the participants was 22.56 (SD 6.16) years. Most of the study participants were women (182/263, 69.2%). The participants were predominantly university students (227/263, 86.3%), with 74.9% (197/263) being undergraduates. Among the 197 undergraduate students, 11.8% (31/197) were in year 1, 17.1% (45/197) in year 2, 22.8% (60/197) in year 3, 20.5% (54/197) in year 4, 1.9% (5/197) in year 5, and 0.8% (2/197) in year 6. The other detailed demographics and baseline characteristics of the participants are presented in Table 1.

Across the four conditions, significant gender differences were found (*χ*^2^_3_=10.0; *P*=.02). The *control* condition had a higher women-to-men ratio than the other 3 conditions. A significant difference in age across conditions was also found in the 1-way ANOVA (*F*_3,259_=3.28; *P*=.02). A correlation analysis was performed to investigate whether gender and age were correlated with mental illness stigma outcomes. Age was not correlated with any of the outcomes, including public stigma (*r*=−0.02; *P*=.81), microaggression (*r*=−0.02; *P*=.74), and social distance (*r*=0.08; *P*=.18). Gender was weakly correlated with public stigma (*r*=−0.14; *P*=.02) and microaggression (*r*=−0.12; *P*=.05) but not with social distance (*r*=−0.01; *P*=.84). No other significant differences in demographic characteristics were found. In terms of participants’ previous experiences with mental illness, no significant differences were found across the conditions. Given the baseline difference in gender across conditions, gender was included as a covariate in subsequent analyses.

**Table 1:** Baseline characteristics across conditions.

Characteristics		*Combo* condition^a^ (n=67)	*Stigma* condition^b^ (n=65)	*Interact* condition^c^ (n=64)	*Control* condition^d^ (n=67)
Age (years), mean (SD)		22.49 (6.33)	24.49 (8.34)	21.89 (3.95)	21.39 (4.78)
**Gender, n (%)**					
	Men	27 (40)	23 (35)	20 (31)	11 (16)
	Women	40 (60)	42 (65)	44 (69)	56 (84)
**Student: current educational level, n (%)**					
	Diploma, certificate, or associate degree	1 (2)	3 (5)	0 (0)	3 (5)
	Bachelor’s degree	52 (78)	42 (65)	48 (75)	55 (82)
	Master’s degree	4 (6)	6 (9)	9 (14)	2 (3)
	Doctoral degree	0 (0)	1 (2)	0 (0)	1 (2)
**Nonstudent: educational attainment, n (%)**					
	Secondary (form 1-6 or 7)	2 (3)	4 (6)	0 (0)	0 (0)
	Diploma, certificate, or associate degree	3 (5)	0 (0)	0 (0)	1 (2)
	Bachelor’s degree	3 (5)	3 (5)	4 (6)	5 (8)
	Master’s degree	2 (3)	5 (8)	2 (3)	0 (0)
	Doctoral degree	0 (0)	1 (2)	1 (2)	0 (0)
**Religion, n (%)**					
	No religion	53 (79)	52 (80)	49 (77)	43 (64)
	Buddhism	0 (0)	1 (2)	1 (2)	1 (2)
	Catholicism	0 (0)	1 (2)	1 (2)	2 (3)
	Christianity	13 (19)	9 (14)	13 (20)	20 (30)
	Taoism	1 (2)	2 (3)	0 (0)	0 (0)
	Others	0 (0)	0 (0)	0 (0)	1 (2)
**Sexual orientation, n (%)**					
	Heterosexual	64 (96)	56 (86)	59 (92)	65 (97)
	Homosexual	0 (0)	6 (9)	1 (2)	0 (0)
	Bisexual	3 (5)	2 (3)	1 (2)	0 (0)
	Pansexual	0 (0)	0 (0)	1 (2)	1 (2)
	Others	0 (0)	1 (2)	2 (3)	1 (2)
Level of previous contact with people having a mental illness, mean (SD)		2.52 (1.26)	2.43 (1.00)	2.20 (0.93)	2.22 (1.00)

^a^Interactivity present; stigma content present.

^b^Interactivity absent; stigma content present.

^c^Interactivity present; stigma content absent.

^d^Interactivity absent; stigma content absent.

### Mental Illness Stigma Measures

#### Public Stigma Toward People With Mental Illness

Results from the repeated measures ANOVA indicated a significant time×condition effect (*P*=.002; η^2^=0.04), and a post hoc analysis was conducted. In the *combo* condition, public stigma significantly decreased from baseline to after the assessment (mean difference 0.61, 95% CI 0.49-0.74; *P*<.001; η^2^=0.37), and the decrease was maintained at the 1-week follow-up (mean difference 0.53, 95% CI 0.37-0.69; *P*<.001; η^2^=0.37). In the *stigma* condition, public stigma also significantly decreased from baseline to after the assessment (mean difference 0.42, 95% CI 0.30-0.55; *P*<.001; η^2^=0.22), and the decrease was maintained at the 1-week follow-up (mean difference 0.34, 95% CI 0.18-0.50; *P*<.001; η^2^=0.22). In the *interact* condition, public stigma significantly decreased from baseline to after the assessment (mean difference 0.14, 95% CI 0.02-0.26; *P*=.02; η^2^=0.03), but the effect was not sustained at the 1-week follow-up (mean difference 0.12, 95% CI −0.04 to 0.28; *P*=.22; η^2^=0.03). In the *control* condition, the effect was not significant from baseline to after the assessment (mean difference 0.07, 95% CI −0.06 to 0.20; *P*=.56; η^2^=0.01) and from baseline to 1-week follow-up (mean difference 0.09, 95% CI −0.08 to 0.26; *P*=.57; η^2^=0.01). In terms of mean difference values, the results indicated that the effect was the strongest in the *combo* condition, followed by the *stigma* and *interact* conditions. An additional post hoc analysis was carried out to compare *combo* and *stigma* conditions; the interaction effect between interactivity and stigma content was not significant (*P*=.09).

#### Microaggression

The results showed a significant time×condition effect (*P*<.001; η^2^=0.06), and a post hoc analysis was carried out. Microaggression significantly decreased from baseline to after the assessment in both the *combo* (mean difference 0.34, 95% CI 0.25-0.42; *P*<.001; η^2^=0.31) and *stigma* (mean difference 0.28, 95% CI 0.19-0.36; *P*<.001; η^2^=0.24) conditions. The effects were sustained and strengthened at the 1-week follow-up in both conditions (*combo*: mean difference 0.39, 95% CI 0.29-0.49; *P*<.001; η^2^=0.31; *stigma*: mean difference 0.33, 95% CI 0.23-0.43; *P*<.001; η^2^=0.24). In the *interact* condition, the effect was not significant from baseline to after the assessment (mean difference 0.03, 95% CI −0.05 to 0.12; *P*=.99; η^2^=0.01) and from baseline to 1-week follow-up (mean difference 0.06, 95% CI −0.04 to 0.16; *P*=.40; η^2^=0.01). In the *control* condition, the effect was also not significant from baseline to after the assessment (mean difference 0.03, 95% CI −0.06 to 0.12; *P*=.99; η^2^=0.01) and from baseline to 1-week follow-up (mean difference −0.04, 95% CI −0.15 to 0.07; *P*=.99; η^2^=0.01). The results indicated that the effect of the *combo* condition was stronger than that of the *stigma* condition in terms of mean difference values. No significant interaction effect between interactivity and stigma content was found (*P*=.58) after performing an additional post hoc analysis to compare the *combo* and *stigma* conditions.

#### Social Distance From People With Mental Illness

The results showed a nonsignificant time×condition effect (*P*=.25; η^2^=0.02). The additional post hoc analysis comparing the *combo* and *stigma* conditions showed no significant interaction effect between interactivity and stigma content (*P*=.46). The details of the repeated measures ANOVA are shown in Table 2.

**Table 2:** Imputed values of means and SDs across conditions.

			*Combo* condition^a^ (n=66), mean (SD)		*Stigma* condition^b^ (n=65), mean (SD)		*Interact* condition^c^ (n=64), mean (SD)		*Control* condition^d^ (n=67), mean (SD)
**Public stigma**									
	Baseline	2.85 (0.66)		2.68 (0.75)		2.83 (0.72)		2.81 (0.72)	
	After the experiment	2.24 (0.66)		2.25 (0.73)		2.69 (0.80)		2.73 (0.75)	
	1-week follow-up	2.33 (0.78)		2.33 (0.74)		2.71 (0.74)		2.75 (0.72)	
**Microaggression**									
	Baseline	2.45 (0.37)		2.44 (0.36)		2.46 (0.32)		2.41 (0.38)	
	After the experiment	2.13 (0.45)		2.17 (0.44)		2.43 (0.37)		2.39 (0.42)	
	1-week follow-up	2.06 (0.43)		2.12 (0.49)		2.40 (0.33)		2.45 (0.40)	
**Social distance**									
	Baseline	2.39 (0.48)		2.28 (0.50)		2.35 (0.49)		2.38 (0.51)	
	After the experiment	1.98 (0.57)		1.97 (0.51)		2.36 (0.48)		2.35 (0.51)	
	1-week follow-up	2.13 (0.53)		2.07 (0.61)		2.41 (0.48)		2.40 (0.53)	

^a^Interactivity present; stigma content present.

^b^Interactivity absent; stigma content present.

^c^Interactivity present; stigma content absent.

^d^Interactivity absent; stigma content absent.

### Mediating Analysis

**Figure 2:**
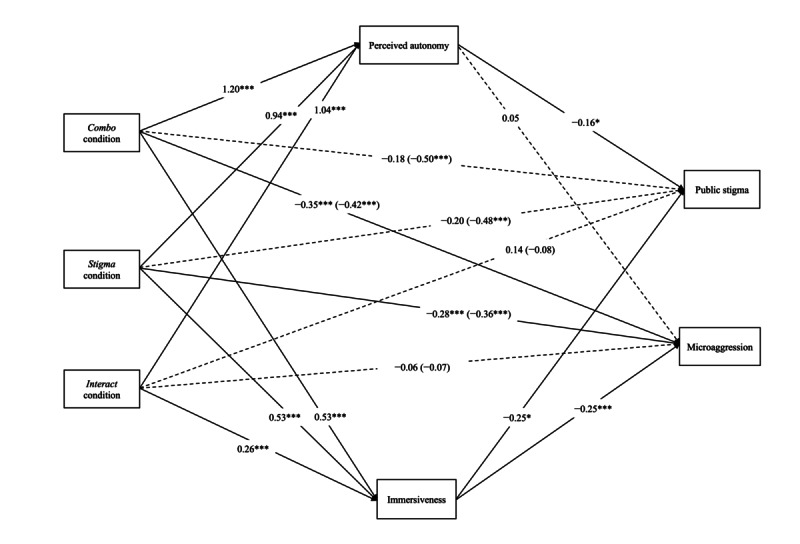
A mediation model of perceived autonomy and immersiveness among conditions. The numbers in brackets denote the total effect, solid lines indicate statistically significant paths, and dotted lines denote nonsignificant paths. Only the main variable is included in the figure for simplicity. *Combo* condition: interactivity present; stigma content present. *Interact* condition: interactivity present; stigma content absent. *Stigma* condition: interactivity absent; stigma content present. *P<.05, **P<.01, ***P<.001.

To compare the mediation effect of perceived autonomy and immersiveness between conditions with public stigma and microaggression, mediation analyses were performed by incorporating both perceived autonomy and immersiveness into PROCESS model 4. Table 3 shows the unstandardized and standardized factor loadings for the model. A mediation model of perceived autonomy and immersiveness between conditions with public stigma and microaggression is shown in Figure 2. Mediation analysis for social distance was not conducted, because no interaction effect was observed in the social distance across conditions.

We observed significant indirect effects of the *combo* (b=−0.19, bias-corrected and accelerated [BCa] CI −0.36 to −0.03), *stigma* (b=−0.15, BCa CI −0.29 to −0.02) and *interact* (b=−0.16, BCa CI −0.32 to −0.02) conditions on public stigma through perceived autonomy. The nonsignificant indirect effects of the *combo* (b=−0.13, BCa CI −0.30 to 0), *stigma* (b=−0.13, BCa CI −0.30 to 0), and *interact* (b=−0.07, BCa CI −0.16 to 0) conditions on public stigma through immersiveness were observed. The results showed that perceived autonomy was a significant mediator between the conditions and public stigma.

We observed nonsignificant indirect effects of the *combo* (b=0.07, BCa CI −0.03 to 0.17), *stigma* (b=0.05, BCa CI −0.03 to 0.14), and *interact* (b=0.06, BCa CI −0.03 to 0.15) conditions on microaggression through perceived autonomy. The indirect effects of the *combo* (b=−0.13, BCa CI −0.23 to −0.06), *stigma* (b=−0.13, BCa CI −0.23 to −0.05), and *interact* (b=−0.07, BCa CI −0.13 to −0.02) conditions on microaggression through immersiveness was significant. The results showed that immersiveness was a significant mediator between the conditions and microaggression.

**Table 3:** Unstandardized and standardized parameter estimates for the hypothesized model.

Parameter estimates (structural model)	Unstandardized B (SE)	Standardized *β*	*t* value (df)	*P* value
*Combo*^a^→perceived autonomy	1.20 (0.13)	1.42	9.53 (257)	<.001
*Combo*→immersiveness	0.53 (0.07)	1.21	7.81 (257)	<.001
*Combo*→public stigma	−0.18 (0.15)	−.23	−1.18 (255)	.24
*Combo*→microaggression	−0.35 (0.09)	−.78	−4.07 (255)	<.001
*Stigma*^b^→perceived autonomy	0.94 (0.13)	1.11	7.50 (257)	<.001
*Stigma*→immersiveness	0.53 (0.07)	1.20	7.80 (257)	<.001
*Stigma*→public stigma	−0.20 (0.15)	−.26	−1.37 (255)	.17
*Stigma*→microaggression	−0.28 (0.08)	−.61	−3.33 (255)	<.001
*Interact*^c^→perceived autonomy	1.04 (0.13)	1.23	8.30 (257)	<.001
*Interact*→immersiveness	0.26 (0.07)	.60	3.87 (257)	<.001
*Interact*→public stigma	0.14 (0.14)	.19	1.01 (255)	.31
*Interact*→microaggression	−0.06 (0.08)	−.14	−0.78 (255)	.44
Gender→perceived autonomy	0.25 (0.10)	.14	2.59 (257)	.01
Gender→immersiveness	0.07 (0.05)	.07	1.30 (257)	.19
Gender→public stigma	−0.24 (0.10)	−.14	−2.38 (255)	.02
Gender→microaggression	−0.09 (0.06)	−.09	−1.57 (255)	.12
Perceived autonomy→public stigma	−0.16 (0.07)	−.17	−2.27 (255)	.02
Perceived autonomy→microaggression	0.05 (0.04)	.10	1.39 (255)	.16
Immersiveness→public stigma	−0.25 (0.13)	−.14	−1.97 (255)	.05
Immersiveness→microaggression	−0.25 (0.07)	−.24	−3.46 (255)	<.001

^a^Interactivity present; stigma content present.

^b^Interactivity absent; stigma content present.

^c^Interactivity present; stigma content absent.

## Discussion

### Principal Findings

This study investigated the effect of internet-based storytelling programs with a combination of stigma content and interactivity on mental illness stigma reduction. Multiple forms of mental illness stigma were accounted for, including public stigma toward people with mental illness, microaggression, and social distance from people with mental illness.

The results supported our hypothesis that an internet-based storytelling program with a combination of stigma content and interactivity was able to significantly reduce public stigma and microaggression immediately after the experiment and at the 1-week follow-up assessment. Contrary to our hypothesis, an internet-based storytelling program with a combination of stigma content and interactivity did not significantly reduce the social distance from people with mental illness. In other words, the storytelling program was more effective in improving individuals’ stigmatizing cognitions, sense of personal advocacy [4], and microaggressions centering around their everyday conversations and encounters in daily life [61] than in enhancing their willingness and intention to behaviorally interact with people with mental illness in various life domains [62].

Nevertheless, the results showed that an internet-based storytelling program with stigma content alone could also lead to a reduction in public stigma and microaggression. Comparing the significant effects elicited by the 2 different internet-based storytelling programs, the one with a combination of stigma content and interactivity produced a stronger effect after the assessment and at the 1-week follow-up assessment than that with stigma content only. These results were consistent with the findings of previous studies showing that internet-based stigma reduction interventions with a combination of interactivity and stigma content can lead to more effective stigma reduction [26]. The results reinforce the importance of correcting stigmatizing misperceptions in diminishing stigma [55,63]. Interestingly, the internet-based storytelling program with interactivity only was also found to reduce public stigma after the assessment although the effect could not be maintained after 1 week. This might support our assumption of a positive relationship between interactivity and positive affect and between positive affect and reduced prejudice [37,40]. Further studies are required to examine these relationships.

Moreover, although perceived autonomy could mediate the effect of conditions on public stigma, immersiveness could mediate the effect of conditions on microaggression. In previous findings, perceived autonomy was associated with the endorsement of the selected responses and actions and cognitive changes [46]. This might demonstrate that cognitive changes are essential for reducing public stigma. On the contrary, immersiveness could facilitate cognitive and affective changes [50-52], which might expedite people to have more intended changes in ameliorating everyday microaggressions. Social distance posited the focus on one’s behavioral intention toward people with mental illness in various dimensions [62], which may have more long-term implications to their life domains (eg, friendship and employment); the short-term sense of choice and immersiveness in storytelling programs may not be sufficient to bring about changes in social distance.

In addition, this study showed that a storytelling program with stigmatized content but without interactivity could also enhance perceived autonomy and immersiveness. These findings align with the literature showing that stigma content with storytelling elements, which served as an internet-based contact with people with lived experiences, was effective in inducing immersiveness [24,28,30]. The inducement of perceived autonomy with the story was outstanding, whereas the effect might be due to the application of a conversational storyline, which allowed one to feel like interacting with the protagonist even without choosing responses and actions. Thus, storytelling in the form of conversations alone might already provide an effective means for participants to immerse themselves and feel a sense of agency.

The superior effect of an internet-based storytelling program with a combination of interactivity and stigma content over the one with stigma content only was not explained by the interaction effect between interactivity and stigma content. Stigma content was a more important criterion than interactivity in inducing stigma reduction. The addition of interactivity was only supplementary to boosting the stigma reduction effect. The results confirmed previous suggestions that enhanced motivation through perceived autonomy and immersiveness would lead to enhanced stigma reduction [43,44]. This study provides empirical support for the rationale behind the use of internet-based storytelling programs, especially for those with stigma-related stories and interactivity.

### Limitations and Future Directions

This study has some limitations that deserve attention. First, our sample mainly consisted of young university students. These findings may not be generalizable to other populations. In social marketing, segmentation of our target population is essential. This study showed that internet-based antistigma storytelling programs with interactivity may be an effective tool in reducing mental illness stigma for young, educated people in the community who are comfortable and skillful in accessing information over the internet. Furthermore, because of the homogeneous nature of our sample, moderation analysis was not performed. Future studies should explore possible moderators of the effect of internet-based stigma reduction interventions, such as gender, age, and education level.

Second, this study did not include a long-term follow-up. Only a 1-week follow-up assessment was included to investigate whether the mediation effect could be sustained for a week to draw possible mediating mechanisms behind the effect of internet-based stigma reduction interventions. It could capture the short-term effect of the intervention but was incapable of predicting the long-term maintenance effect of stigma reduction [64]. However, the long-term effect must be tested to provide insights into developing sustainable and effective stigma reduction interventions. It is unknown whether an internet-based storytelling program with a combination of interactivity and stigma content could impose a more prolonged stigma reduction effect when than a program with stigma content only. To examine the stigma reduction effect of various internet-based storytelling programs over time, future research should lengthen its follow-up.

Third, this study lacked behavioral measures but solely used self-report measures that tapped into the thoughts, affect, and behavioral intentions of the participants. Although the measures used have been empirically validated, future studies aiming to examine stigma change should also use behavioral measures, such as the inclusion of offering payment for completing questionnaires and the option of donating the payment to a mental health charity, in addition to self-report measures to bolster the findings.

The necessary intensity of storytelling and interactivity to strengthen the stigma reduction effect should also be investigated. Research has shown that placing individuals in a highly immersive environment might lead to worsening attitudes [65], which varies according to the degree of identification with the embodied target [66]. Therefore, the degree of immersiveness elicited should be considered. In addition, future studies can explore how individual differences, such as the baseline level of empathy, may influence intervention benefits, as internet-based interventions with different adaptations might cater to different segments of the population. People with different levels of dispositional empathy may have variable receptivity to internet-based storytelling programs, and matching their styles with this approach may maximize the outcomes.

Finally, this study suggests that increased perceived autonomy and immersiveness could strengthen the stigma reduction effect in internet-based storytelling programs with interactivity and stigma content. With the advancement in virtual reality technology and the sense of embodiment and story transportation being found to mediate public stigma reduction [67], future studies can consider comparing virtual reality–based and internet-based stigma reduction programs to shed light on which delivery methods are more effective and cost-effective in reducing mental illness stigma.

### Conclusions

In sum, this study showed that internet-based storytelling programs with a combination of interactivity and stigma content are effective in reducing mental illness stigma, and perceived autonomy and immersiveness are significant mechanisms in the stigma reduction process. The findings of this study are encouraging and support the possible mechanisms behind the effects of internet-based storytelling programs. Furthermore, the study upholds the gravity of stigma content, as the strength of interactivity in reducing mental illness stigma can only be manifested in the presence of stigma content. To leverage the power of technology in reducing mental illness stigma, it is paramount for antistigma campaigns to incorporate these active ingredients into the design of antistigma interventions to create low-cost, effective, and scalable internet-based stigma reduction programs.

## Appendix

Multimedia Appendix 1 Screenshots of the four web pages.

Multimedia Appendix 2 CONSORT-eHEALTH checklist (V 1.6.1).

## Abbreviations

BCa: bias-corrected and accelerated
CONSORT: Consolidated Standards of Reporting Trials
