# Supplementary material for: The Effects of Internet-Based Storytelling Programs (Amazing Adventure Against Stigma) in Reducing Mental Illness Stigma With Mediation by Interactivity and Stigma Content: Randomized Controlled Trial
Source: J Med Internet Res. 2022 Aug 12;24(8):e37973. doi: 10.2196/37973 (PMC9419045; doi:10.2196/37973)

Multimedia Appendix 1: screenshots of the four web pages.

Combo condition: interactivity present, stigma content present.
Website: https://antistigma.psy.cuhk.edu.hk
Interactivity present: participants could choose their actions and responses in the web page.
Stigma content present: the web page illustrated the person with lived experience of mental illness accompanied participants to visualize his microaggressive encounters in various life domains (e.g., work, family, social circle) and the public's misunderstanding towards mental illness.

(1) First page: allow participants to enter their names.	(2) Allow participants to choose their avatars.
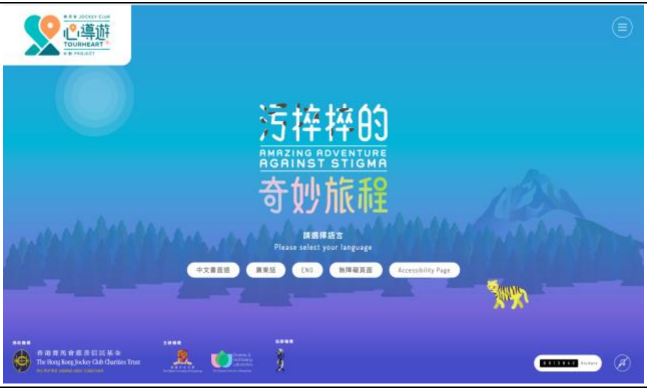	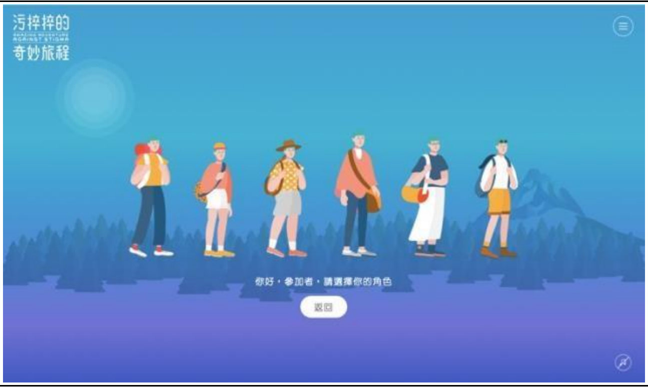
(3) Content about the encounters of the person with lived experience of mental illness: allow participants to choose the sequence of reading.	(4) Allow participants to choose a response out of the three given choices of response.
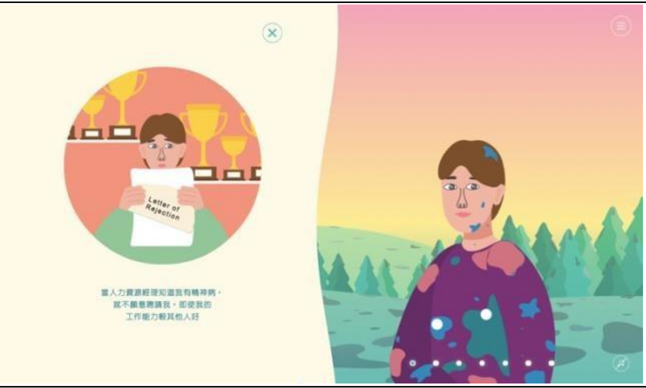	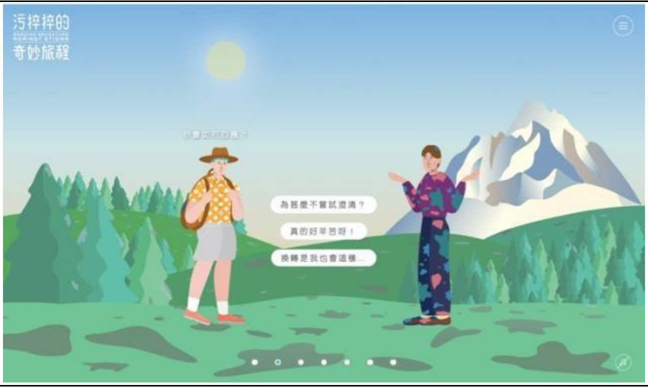
(5) Content about mental illness stigma and Question and Answer session: allow participants to choose the "true" or "false" button towards some common misunderstandings about mental illness, and the respective responses and evidence-based facts aiming to correct the misunderstandings are given according to the button chosen.	(6) Content about microaggression and microaggressive encounters experienced by the host in different settings, including workplace, family and social circle: allow participants to tap "proceed" when they finish reading.
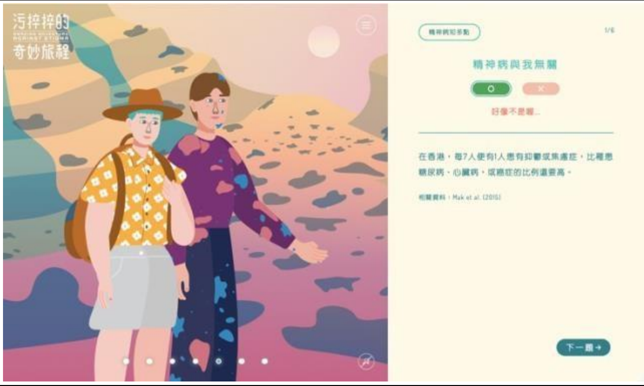	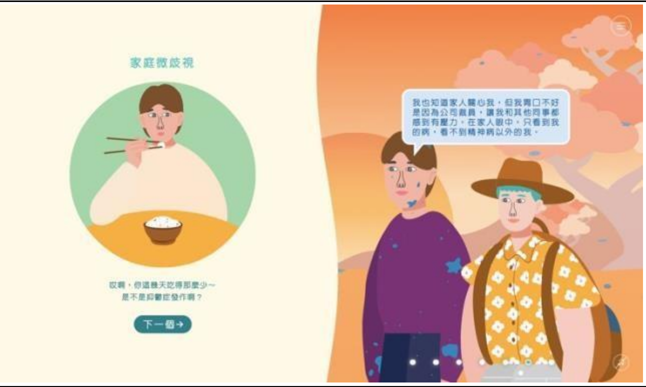

Stigma condition: interactivity absent, stigma content present.
Interactivity absent: participants could not choose their actions and responses in the web page.
Stigma content present: the web page illustrated the person with lived experience of mental illness accompanied participants to visualize his microaggressive encounters in various life domains (e.g., work, family, social circle) and the public's misunderstanding towards mental illness.

(1) First page.	(2) The avatar representing the participant is fixed.
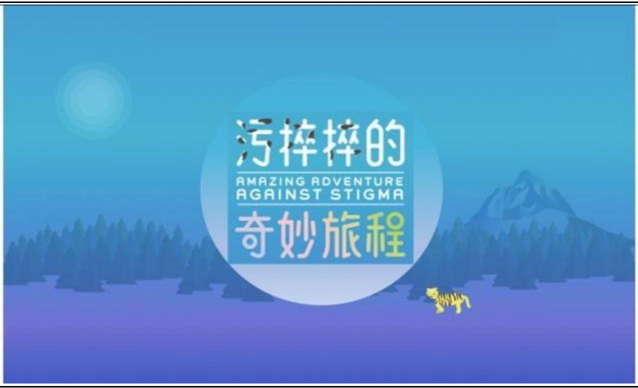	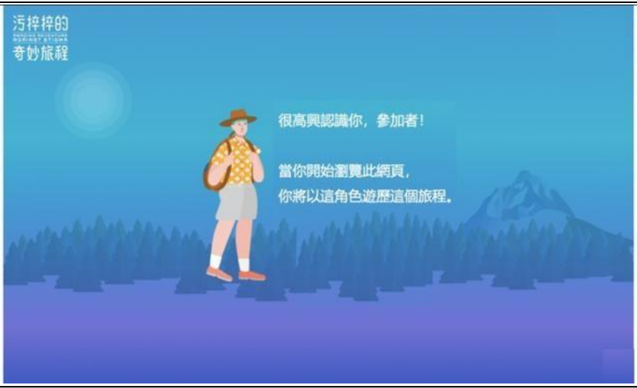
(3) Content about the encounters of the person with lived experience of mental illness with fixed sequence.	(4) No choices of response are given. The conversation between the avatar and the host is preassigned.
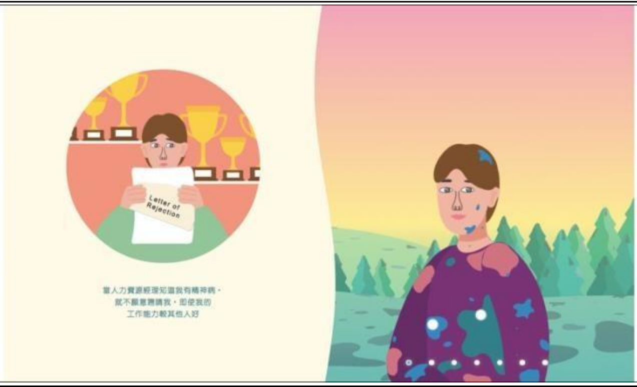	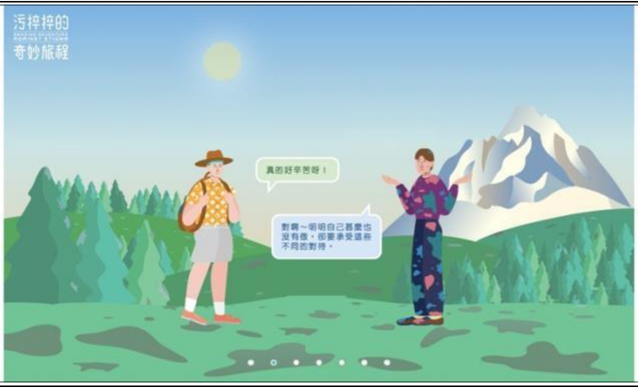
(5) Content about mental illness stigma and Question and Answer session: some common misunderstandings about mental illness are displayed as statements, and the evidence-based facts aiming to correct the misunderstandings are elaborated.	(6) Content about microaggression and microaggressive encounters experienced by the host in different settings, including workplace, family and social circle.
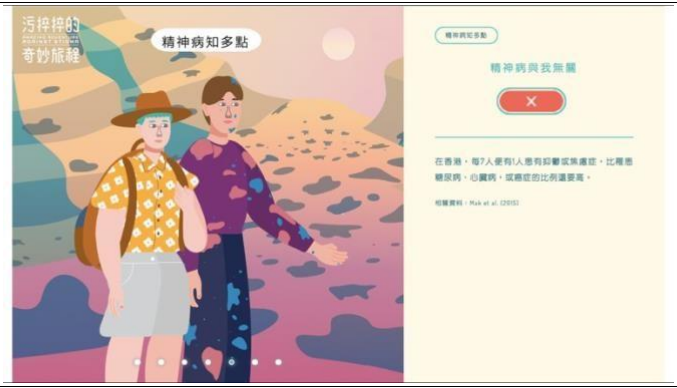	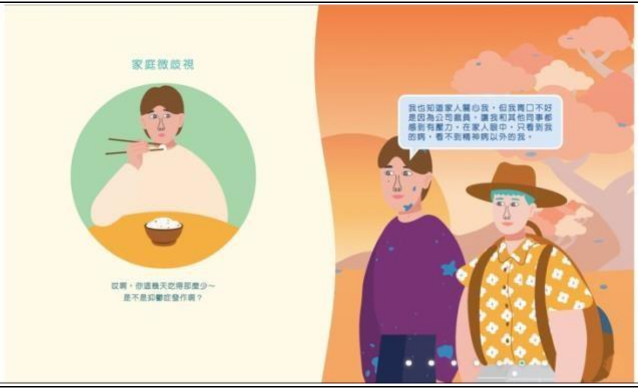

Interact condition: interactivity present, stigma content absent.
Interactivity present: participants could choose their actions and responses in the web page.
Stigma content absent: the web page illustrated a typical day of a person.

(1) First page: introduce to the participants that they would experience the journey as the avatar shown, allow participants to tap "proceed" when they finish reading.

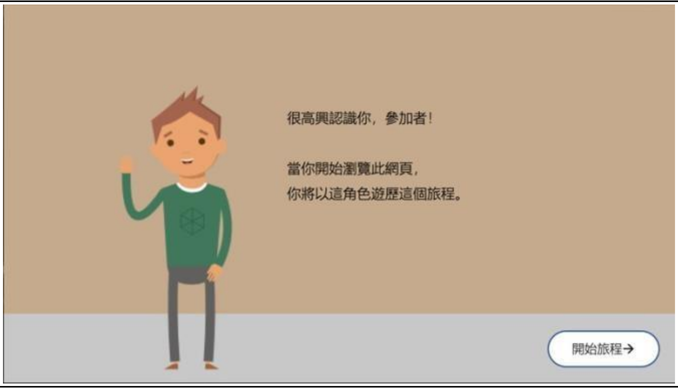

(2) Beginning of the typical day of the avatar: allow participants to tap "proceed" when they finish reading.

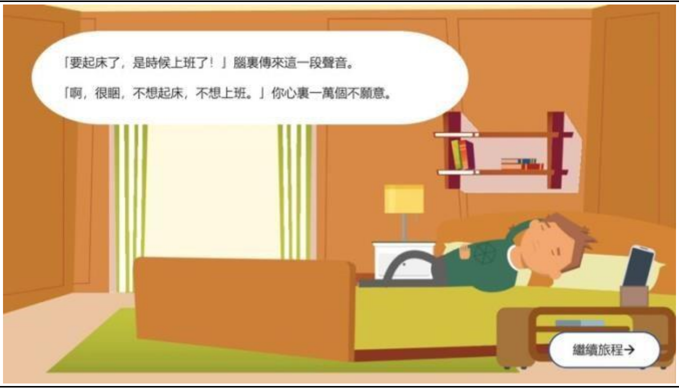

(3) Participants are able to choose the clothes they would like to wear as the avatar.

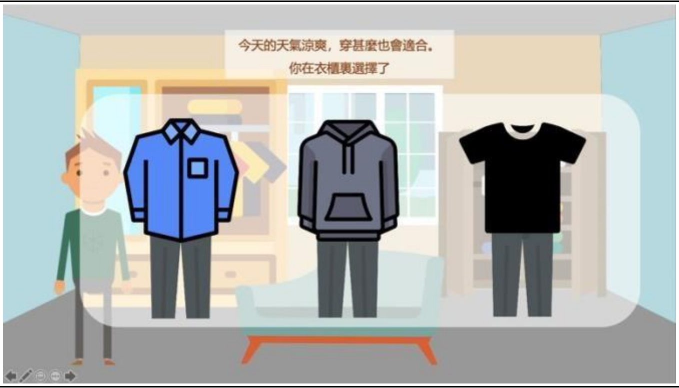

(4) Participants are able to choose the transportation they would like to take as the avatar.

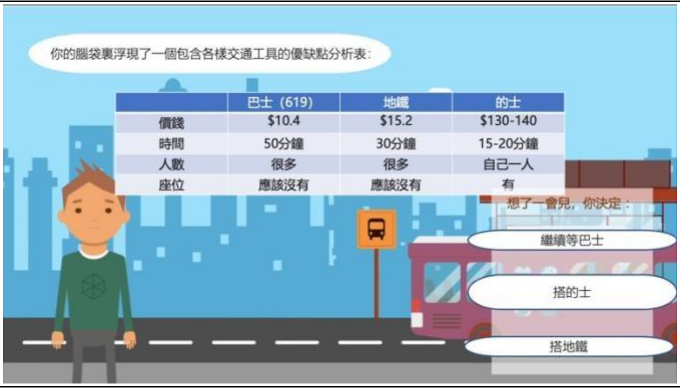

(5) Content about different kinds of rock: allow participants to tap "proceed" when they finish reading.

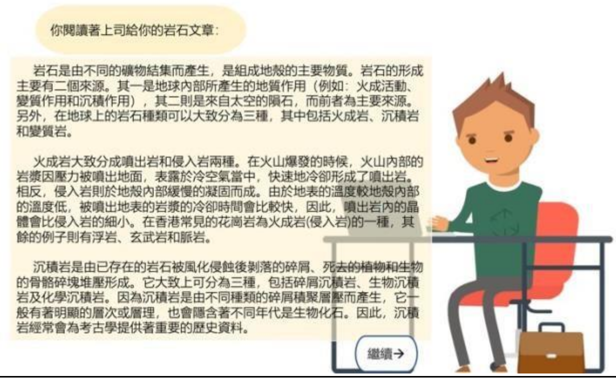

(6) Question and Answer session: allow participants to choose the "true" or "false" button towards some statements about plants, and the respective responses and facts are given according to the button chosen.

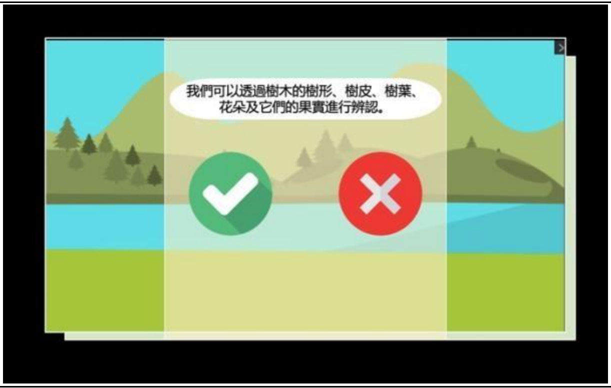

Control condition: interactivity absent, stigma content absent.
Interactivity absent: participants could not choose their actions and responses in the web page.
Stigma content absent: the web page illustrated a typical day of a person.

(1) First page: introduce to the participants that they would experience the journey as the avatar shown.

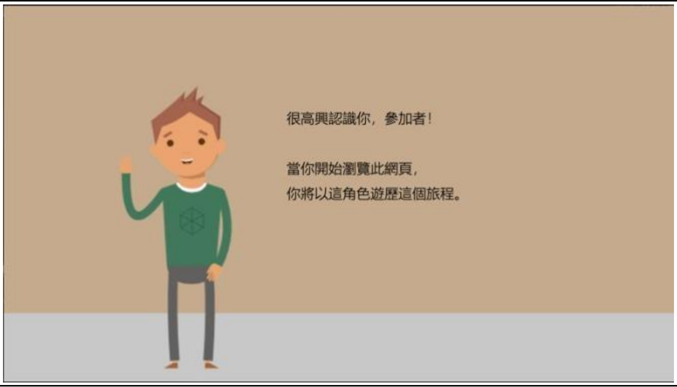

(2) Beginning of the typical day of the avatar.

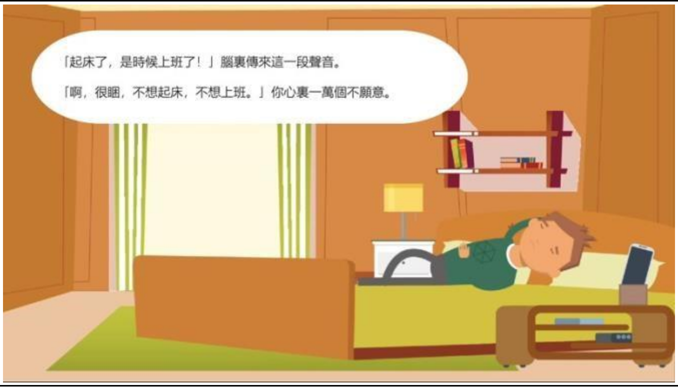

(3) The clothes worn by the avatar are fixed.

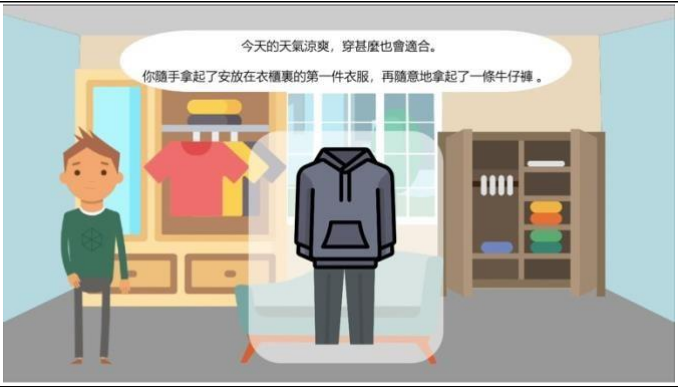

(4) The transportation taken by the avatar is fixed.

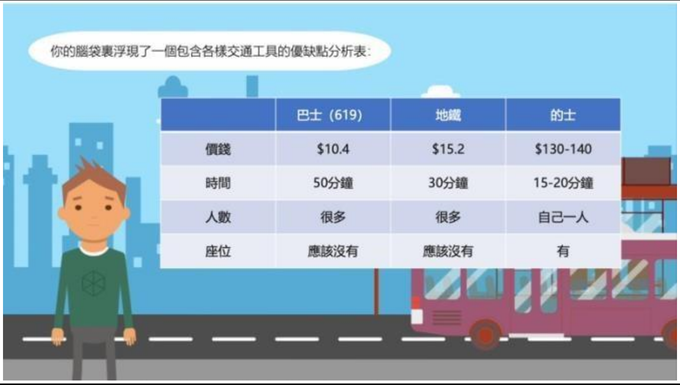

(5) Content about different kinds of rock.

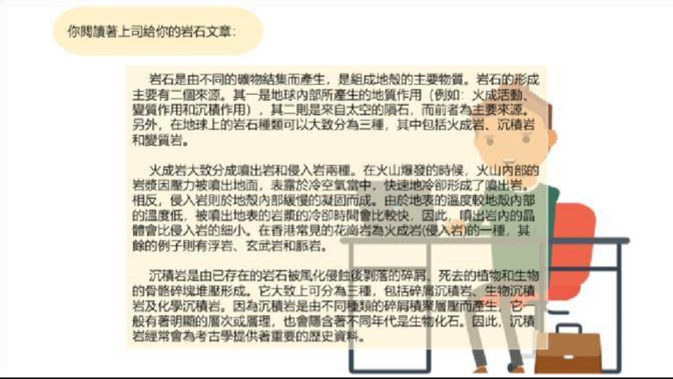

(6) Question and Answer session: some statements about plants are displayed, and the facts aiming to correct the misunderstandings are elaborated.

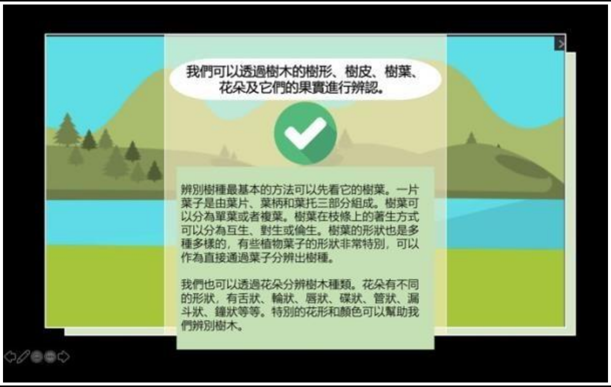

Supplement: Multimedia Appendix 1 [file jmir_v24i8e37973_app1.pdf]
