# Supplementary material for: The Effects of Internet-Based Storytelling Programs (Amazing Adventure Against Stigma) in Reducing Mental Illness Stigma With Mediation by Interactivity and Stigma Content: Randomized Controlled Trial
Source: J Med Internet Res. 2022 Aug 12;24(8):e37973. doi: 10.2196/37973 (PMC9419045; doi:10.2196/37973)
Supplement: Multimedia Appendix 2 [file jmir_v24i8e37973_app2.pdf]

CONSORT-EHEALTH (V 1.6.1) - Submission/Publication Form

The CONSORT-EHEALTH checklist is intended for authors of randomized trials evaluating web-based and Internet-based applications/interventions, including mobile interventions, electronic games (incl multiplayer games), social media, certain telehealth applications, and other interactive and/or networked electronic applications. Some of the items (e.g. all subitems under item 5 - description of the intervention) may also be applicable for other study designs.

The goal of the CONSORT EHEALTH checklist and guideline is to be
a) a guide for reporting for authors of RCTs,
b) to form a basis for appraisal of an ehealth trial (in terms of validity)

CONSORT-EHEALTH items/subitems are MANDATORY reporting items for studies published in the Journal of Medical Internet Research and other journals / scientific societies endorsing the checklist.

Items numbered 1., 2., 3., 4a., 4b etc are original CONSORT or CONSORT-NPT (non-pharmacologic treatment) items.
Items with Roman numerals (i., ii, iii, iv etc.) are CONSORT-EHEALTH extensions/clarifications.

As the CONSORT-EHEALTH checklist is still considered in a formative stage, we would ask that you also RATE ON A SCALE OF 1-5 how important/useful you feel each item is FOR THE PURPOSE OF THE CHECKLIST and reporting guideline (optional).

Mandatory reporting items are marked with a red *.
In the textboxes, either copy & paste the relevant sections from your manuscript into this form - please include any quotes from your manuscript in QUOTATION MARKS,
or answer directly by providing additional information not in the manuscript, or elaborating on why the item was not relevant for this study.

YOUR ANSWERS WILL BE PUBLISHED AS A SUPPLEMENTARY FILE TO YOUR PUBLICATION IN JMIR AND ARE CONSIDERED PART OF YOUR PUBLICATION (IF ACCEPTED).
Please fill in these questions diligently. Information will not be copyedited, so please use proper spelling and grammar, use correct capitalization, and avoid abbreviations.

DO NOT FORGET TO SAVE AS PDF _AND_ CLICK THE SUBMIT BUTTON SO YOUR ANSWERS ARE IN OUR DATABASE !!!

Citation Suggestion (if you append the pdf as Appendix we suggest to cite this paper in the caption):
Eysenbach G, CONSORT-EHEALTH Group
CONSORT-EHEALTH: Improving and Standardizing Evaluation Reports of Web-based and Mobile Health Interventions
J Med Internet Res 2011;13(4):e126
URL: http://www.jmir.org/2011/4/e126/
doi: 10.2196/jmir.1923

PMID: 22209829

tiffanyfonghc@gmail.com (not shared) Switch accounts

Resubmit to save

*Required

Your name *

First Last

Hiu Ching Tiffany Fong

Primary Affiliation (short), City, Country *

University of Toronto, Toronto, Canada

The Chinese University of Hong Kong, Hong Kc

Your e-mail address *

abc@gmail.com

tiffanyfonghc@gmail.com

Title of your manuscript *

Provide the (draft) title of your manuscript.

Effects of Internet-based Storytelling Programs in Reducing Mental Illness Stigma with Mediation by Interactivity and Stigma Content: A Randomized Controlled Trial

Name of your App/Software/Intervention *

If there is a short and a long/alternate name, write the short name first and add the long name in brackets.

The Amazing Adventure Against Stigma websi

Evaluated Version (if any)

e.g. "V1", "Release 2017-03-01", "Version 2.0.27913"

Your answer

Language(s) *

What language is the intervention/app in? If multiple languages are available, separate by comma (e.g. "English, French")

Chinese

URL of your Intervention Website or App

e.g. a direct link to the mobile app on app in appstore (itunes, Google Play), or URL of the website. If the intervention is a DVD or hardware, you can also link to an Amazon page.

https://antistigma.psy.cuhk.edu.hk/

URL of an image/screenshot (optional)

Your answer

Accessibility *

Can an enduser access the intervention presently?

- (●) access is free and open
- ( ) access only for special usergroups, not open
- ( ) access is open to everyone, but requires payment/subscription/in-app purchases
- ( ) app/intervention no longer accessible
- ( ) Other:

Primary Medical Indication/Disease/Condition *

e.g. "Stress", "Diabetes", or define the target group in brackets after the condition, e.g. "Autism (Parents of children with)", "Alzheimers (Informal Caregivers of)"

Mental health stigma

Primary Outcomes measured in trial *

comma-separated list of primary outcomes reported in the trial

Public stigma towards people with mental illne

Secondary/other outcomes

Are there any other outcomes the intervention is expected to affect?

Your answer

Recommended "Dose" *

What do the instructions for users say on how often the app should be used?

- ○ Approximately Daily
- ○ Approximately Weekly
- ○ Approximately Monthly
- ○ Approximately Yearly
- ◉ "as needed"
- ○ Other:

Approx. Percentage of Users (starters) still using the app as recommended after 3 months *

- (x) unknown / not evaluated
- ( ) 0-10%
- ( ) 11-20%
- ( ) 21-30%
- ( ) 31-40%
- ( ) 41-50%
- ( ) 51-60%
- ( ) 61-70%
- ( ) 71%-80%
- ( ) 81-90%
- ( ) 91-100%
- ( ) Other:

Overall, was the app/intervention effective? *

- ( ) yes: all primary outcomes were significantly better in intervention group vs control
- (x) partly: SOME primary outcomes were significantly better in intervention group vs control
- ( ) no statistically significant difference between control and intervention
- ( ) potentially harmful: control was significantly better than intervention in one or more outcomes
- ( ) inconclusive: more research is needed
- ( ) Other:

Article Preparation Status/Stage *

At which stage in your article preparation are you currently (at the time you fill in this form)

- ○ not submitted yet - in early draft status
- ○ not submitted yet - in late draft status, just before submission
- ○ submitted to a journal but not reviewed yet
- ◉ submitted to a journal and after receiving initial reviewer comments
- ○ submitted to a journal and accepted, but not published yet
- ○ published
- ○ Other:

Journal *

If you already know where you will submit this paper (or if it is already submitted), please provide the journal name (if it is not JMIR, provide the journal name under "other")

- ○ not submitted yet / unclear where I will submit this
- ◉ Journal of Medical Internet Research (JMIR)
- ○ JMIR mHealth and UHealth
- ○ JMIR Serious Games
- ○ JMIR Mental Health
- ○ JMIR Public Health
- ○ JMIR Formative Research
- ○ Other JMIR sister journal
- ○ Other:

Is this a full powered effectiveness trial or a pilot/feasibility trial? *

- ○ Pilot/feasibility
- ◉ Fully powered

Manuscript tracking number *

If this is a JMIR submission, please provide the manuscript tracking number under "other" (The ms tracking number can be found in the submission acknowledgement email, or when you login as author in JMIR. If the paper is already published in JMIR, then the ms tracking number is the four-digit number at the end of the DOI, to be found at the bottom of each published article in JMIR)

- ○ no ms number (yet) / not (yet) submitted to / published in JMIR
- ◉ Other: 37973

TITLE AND ABSTRACT

1a) TITLE: Identification as a randomized trial in the title

1a) Does your paper address CONSORT item 1a? *

I.e does the title contain the phrase "Randomized Controlled Trial"? (if not, explain the reason under "other")

- ◉ yes
- ○ Other:

1a-i) Identify the mode of delivery in the title

Identify the mode of delivery. Preferably use “web-based” and/or “mobile” and/or “electronic game” in the title. Avoid ambiguous terms like “online”, “virtual”, “interactive”. Use “Internet-based” only if Intervention includes non-web-based Internet components (e.g. email), use “computer-based” or “electronic” only if offline products are used. Use “virtual” only in the context of “virtual reality” (3-D worlds). Use “online” only in the context of “online support groups”. Complement or substitute product names with broader terms for the class of products (such as “mobile” or “smart phone” instead of “iphone”), especially if the application runs on different platforms.

	1	2	3	4	5	
subitem not at all important	○	○	○	◉	○	essential

Clear selection

Does your paper address subitem 1a-i? *

Copy and paste relevant sections from manuscript title (include quotes in quotation marks "like this" to indicate direct quotes from your manuscript), or elaborate on this item by providing additional information not in the ms, or briefly explain why the item is not applicable/relevant for your study

"Internet-based Storytelling Programs"

1a-ii) Non-web-based components or important co-interventions in title

Mention non-web-based components or important co-interventions in title, if any (e.g., “with telephone support”).

	1	2	3	4	5	
subitem not at all important	○	○	◉	○	○	essential

Clear selection

Does your paper address subitem 1a-ii?

Copy and paste relevant sections from manuscript title (include quotes in quotation marks "like this" to indicate direct quotes from your manuscript), or elaborate on this item by providing additional information not in the ms, or briefly explain why the item is not applicable/relevant for your study

The non-web based components are involved in the INTERACT and CONTROL condition only.

1a-iii) Primary condition or target group in the title

Mention primary condition or target group in the title, if any (e.g., "for children with Type I Diabetes") Example: A Web-based and Mobile Intervention with Telephone Support for Children with Type I Diabetes: Randomized Controlled Trial

	1	2	3	4	5	
subitem not at all important	○	○	○	○	◉	essential

Clear selection

Does your paper address subitem 1a-iii? *

Copy and paste relevant sections from manuscript title (include quotes in quotation marks "like this" to indicate direct quotes from your manuscript), or elaborate on this item by providing additional information not in the ms, or briefly explain why the item is not applicable/relevant for your study

"Mental Illness Stigma", "Interactivity", "Stigma Content"

1b) ABSTRACT: Structured summary of trial design, methods, results, and conclusions

NPT extension: Description of experimental treatment, comparator, care providers, centers, and blinding status.

1b-i) Key features/functionalities/components of the intervention and comparator in the METHODS section of the ABSTRACT

Mention key features/functionalities/components of the intervention and comparator in the abstract. If possible, also mention theories and principles used for designing the site. Keep in mind the needs of systematic reviewers and indexers by including important synonyms. (Note: Only report in the abstract what the main paper is reporting. If this information is missing from the main body of text, consider adding it)

	1	2	3	4	5	
subitem not at all important	○	○	○	◉	○	essential

Clear selection

Does your paper address subitem 1b-i? *

Copy and paste relevant sections from the manuscript abstract (include quotes in quotation marks "like this" to indicate direct quotes from your manuscript), or elaborate on this item by providing additional information not in the ms, or briefly explain why the item is not applicable/relevant for your study

"This study compared the effects of four storytelling websites varied on levels of interactivity and stigma content using an experimental design. Specifically, the conditions included an interactive website with stigma-related content (COMBO condition), a non-interactive website with stigma content (STIGMA condition), an interactive website without stigma- related content (INTERACT condition) and a non-interactive website without stigma-related content (CONTROL condition). Participants were recruited via mass emails to all students and staff of a public university and social networking sites. Eligible participants were randomized into four conditions: COMBO (n=67), STIGMA (n=65), INTERACT (n=64) or CONTROL (n=67). Participants viewed the respective Web page at their own pace. Public stigma, microaggression, and social distance were measured online at pre-experiment, post-experiment, and 1-week follow-up. Perceived autonomy and immersiveness as mediators were assessed at post-experiment."

1b-ii) Level of human involvement in the METHODS section of the ABSTRACT

Clarify the level of human involvement in the abstract, e.g., use phrases like "fully automated" vs. "therapist/nurse/care provider/physician-assisted" (mention number and expertise of providers involved, if any). (Note: Only report in the abstract what the main paper is reporting. If this information is missing from the main body of text, consider adding it)

	1	2	3	4	5	
subitem not at all important	○	○	●	○	○	essential

Clear selection

Does your paper address subitem 1b-ii?

Copy and paste relevant sections from the manuscript abstract (include quotes in quotation marks "like this" to indicate direct quotes from your manuscript), or elaborate on this item by providing additional information not in the ms, or briefly explain why the item is not applicable/relevant for your study

"Participants viewed the respective Web page at their own pace."

1b-iii) Open vs. closed, web-based (self-assessment) vs. face-to-face assessments in the METHODS section of the ABSTRACT

Mention how participants were recruited (online vs. offline), e.g., from an open access website or from a clinic or a closed online user group (closed usergroup trial), and clarify if this was a purely web-based trial, or there were face-to-face components (as part of the intervention or for assessment). Clearly say if outcomes were self-assessed through questionnaires (as common in web-based trials). Note: In traditional offline trials, an open trial (open-label trial) is a type of clinical trial in which both the researchers and participants know which treatment is being administered. To avoid confusion, use “blinded” or “unblinded” to indicated the level of blinding instead of “open”, as “open” in web-based trials usually refers to “open access” (i.e. participants can self-enrol). (Note: Only report in the abstract what the main paper is reporting. If this information is missing from the main body of text, consider adding it)

	1	2	3	4	5	
subitem not at all important	○	◉	○	○	○	essential

Clear selection

Does your paper address subitem 1b-iii?

Copy and paste relevant sections from the manuscript abstract (include quotes in quotation marks "like this" to indicate direct quotes from your manuscript), or elaborate on this item by providing additional information not in the ms, or briefly explain why the item is not applicable/relevant for your study

"Participants were recruited via mass emails to all students and staff of a public university and social networking sites."
"Participants viewed the respective Web page at their own pace."
"Public stigma, microaggression, and social distance were measured online at pre-experiment, post-experiment, and 1-week follow-up. Perceived autonomy and immersiveness as mediators were assessed at post-experiment."

1b-iv) RESULTS section in abstract must contain use data

Report number of participants enrolled/assessed in each group, the use/uptake of the intervention (e.g., attrition/adherence metrics, use over time, number of logins etc.), in addition to primary/secondary outcomes. (Note: Only report in the abstract what the main paper is reporting. If this information is missing from the main body of text, consider adding it)

	1	2	3	4	5	
subitem not at all important	○	○	○	◉	○	essential

Clear selection

Does your paper address subitem 1b-iv?

Copy and paste relevant sections from the manuscript abstract (include quotes in quotation marks "like this" to indicate direct quotes from your manuscript), or elaborate on this item by providing additional information not in the ms, or briefly explain why the item is not applicable/relevant for your study

"Both COMBO (n=66) and STIGMA (n=65) conditions were efficacious in reducing public stigma and microaggression towards people with mental illness at post- and 1-week follow-up. However, none of the conditions had significant time x condition effects in reducing social distance from people with mental illness. INTERACT condition (n=64) can significantly reduce public stigma at post- but not 1-week follow-up. CONTROL condition (n=67) was not significant in reducing all mental illness stigma outcomes. Perceived autonomy was found to mediate the effect of public stigma, and immersiveness mediated the effect of microaggression."

1b-v) CONCLUSIONS/DISCUSSION in abstract for negative trials

Conclusions/Discussions in abstract for negative trials: Discuss the primary outcome - if the trial is negative (primary outcome not changed), and the intervention was not used, discuss whether negative results are attributable to lack of uptake and discuss reasons. (Note: Only report in the abstract what the main paper is reporting. If this information is missing from the main body of text, consider adding it)

	1	2	3	4	5	
subitem not at all important	○	○	○	●	○	essential

Clear selection

Does your paper address subitem 1b-v?

Copy and paste relevant sections from the manuscript abstract (include quotes in quotation marks "like this" to indicate direct quotes from your manuscript), or elaborate on this item by providing additional information not in the ms, or briefly explain why the item is not applicable/relevant for your study

"Internet-based storytelling program with stigma-related content and interactivity elicited the largest effects in stigma reduction, including reductions in public stigma and microaggression, although its difference with Internet-based storytelling programs with stigma-related content only was not statistically significant. In other words, although interactivity could strengthen the stigma reduction effect, stigma-related content was a more critical element than interactivity in reducing stigma. Future stigma reduction efforts should place higher priority in producing effective stigma content in a Web page, followed by considering the value of incorporating interactivity in future Internet-based storytelling programs."

INTRODUCTION

2a) In INTRODUCTION: Scientific background and explanation of rationale

2a-i) Problem and the type of system/solution

Describe the problem and the type of system/solution that is object of the study: intended as stand-alone intervention vs. incorporated in broader health care program? Intended for a particular patient population? Goals of the intervention, e.g., being more cost-effective to other interventions, replace or complement other solutions? (Note: Details about the intervention are provided in “Methods” under 5)

	1	2	3	4	5	
subitem not at all important	○	○	○	◉	○	essential

Clear selection

Does your paper address subitem 2a-i? *

Copy and paste relevant sections from the manuscript (include quotes in quotation marks "like this" to indicate direct quotes from your manuscript), or elaborate on this item by providing additional information not in the ms, or briefly explain why the item is not applicable/relevant for your study

"Mental illness stigma is a globally concerning issue due to its detrimental effects imposed on people with mental illness across various life domains (e.g., education, housing, employment, healthcare) during their recovery and their willingness to seek help [1-3]."
"However, the content and design of these Internet-based stigma reduction programs varied largely and limited efforts have been put to investigate the common factors contributing to their effectiveness."
"Apart from incorporating the critical determinants, namely education and contact, in stigma reduction, many Internet-based interventions have made use of interactivity and storytelling in their designs and have demonstrated positive results in reducing mental illness stigma [24-28]. Yet, the types of interactivity are diverse and it is unknown whether the addition of interactivity induces significant positive attitudinal changes that should be valued."

2a-ii) Scientific background, rationale: What is known about the (type of) system

Scientific background, rationale: What is known about the (type of) system that is the object of the study (be sure to discuss the use of similar systems for other conditions/diagnoses, if appropiate), motivation for the study, i.e. what are the reasons for and what is the context for this specific study, from which stakeholder viewpoint is the study performed, potential impact of findings [2]. Briefly justify the choice of the comparator.

	1	2	3	4	5	
subitem not at all important	○	○	○	◉	○	essential

Clear selection

Does your paper address subitem 2a-ii? *

Copy and paste relevant sections from the manuscript (include quotes in quotation marks "like this" to indicate direct quotes from your manuscript), or elaborate on this item by providing additional information not in the ms, or briefly explain why the item is not applicable/relevant for your study

"Recently, Internet-based programs tackling mental illness stigma have been established across the globe due to their low cost, accessibility, and scalability [12-14]."
"Research has also shown that Internet-based and face-to-face stigma reduction programs are equally effective [20,21]."
"According to the Systemic Thinking Model, in interactive environments, interactivity allows individuals to be the agent and effect physical environmental changes that best align with their thinking needs and flow [36]."
"Research has found interactivity to have a significant role in improving information processing through enhanced motivation, which facilitates stigma reduction [26]. Perceived autonomy and immersiveness have been found to enhance motivation [43,44]"

2b) In INTRODUCTION: Specific objectives or hypotheses

Does your paper address CONSORT subitem 2b? *

Copy and paste relevant sections from the manuscript (include quotes in quotation marks "like this" to indicate direct quotes from your manuscript), or elaborate on this item by providing additional information not in the ms, or briefly explain why the item is not applicable/relevant for your study

"The present experimental study aimed to investigate the effect of Internet-based storytelling programs with the manipulation of stigma-related content and interactivity. In the present study, we hypothesized that an Internet-based storytelling program with a combination of interactivity and stigma content would lead to the most significant reduction in public stigma, microaggression, and social distance from people with mental illnesses, followed by Internet-based storytelling program with stigma content-only and interactivity-only, compared with control. Second, we hypothesized that the effects observed in stigma reduction would be mediated by perceived autonomy and immersiveness due to the presence of interactivity."

METHODS

3a) Description of trial design (such as parallel, factorial) including allocation ratio

Does your paper address CONSORT subitem 3a? *

Copy and paste relevant sections from the manuscript (include quotes in quotation marks "like this" to indicate direct quotes from your manuscript), or elaborate on this item by providing additional information not in the ms, or briefly explain why the item is not applicable/relevant for your study

"Participants were then randomly assigned to one of the four experimental conditions through block randomization."

3b) Important changes to methods after trial commencement (such as eligibility criteria), with reasons

Does your paper address CONSORT subitem 3b? *

Copy and paste relevant sections from the manuscript (include quotes in quotation marks "like this" to indicate direct quotes from your manuscript), or elaborate on this item by providing additional information not in the ms, or briefly explain why the item is not applicable/relevant for your study

There were no changes to methods after trial commencement.

3b-i) Bug fixes, Downtimes, Content Changes

Bug fixes, Downtimes, Content Changes: ehealth systems are often dynamic systems. A description of changes to methods therefore also includes important changes made on the intervention or comparator during the trial (e.g., major bug fixes or changes in the functionality or content) (5-iii) and other “unexpected events” that may have influenced study design such as staff changes, system failures/downtimes, etc. [2].

	1	2	3	4	5	
subitem not at all important	○	◉	○	○	○	essential

Clear selection

Does your paper address subitem 3b-i?

Copy and paste relevant sections from the manuscript (include quotes in quotation marks "like this" to indicate direct quotes from your manuscript), or elaborate on this item by providing additional information not in the ms, or briefly explain why the item is not applicable/relevant for your study

There were no changes after trial commencement.

4a) Eligibility criteria for participants

Does your paper address CONSORT subitem 4a? *

Copy and paste relevant sections from the manuscript (include quotes in quotation marks "like this" to indicate direct quotes from your manuscript), or elaborate on this item by providing additional information not in the ms, or briefly explain why the item is not applicable/relevant for your study

"The study targeted people who were (1) aged 18 years or above, and (2) able to read and understand Chinese."

4a-i) Computer / Internet literacy

Computer / Internet literacy is often an implicit “de facto” eligibility criterion - this should be explicitly clarified.

	1	2	3	4	5	
subitem not at all important	○	○	◉	○	○	essential

Clear selection

Does your paper address subitem 4a-i?

Copy and paste relevant sections from the manuscript (include quotes in quotation marks "like this" to indicate direct quotes from your manuscript), or elaborate on this item by providing additional information not in the ms, or briefly explain why the item is not applicable/relevant for your study

"Recruitment was done through sending mass emails to students and staff at a public university in Hong Kong and by posting advertisements on social media."
As the whole recruitment procedure was done online, participants successful registered were considered as having an adequate level of computer literacy.

4a-ii) Open vs. closed, web-based vs. face-to-face assessments:

Open vs. closed, web-based vs. face-to-face assessments: Mention how participants were recruited (online vs. offline), e.g., from an open access website or from a clinic, and clarify if this was a purely web-based trial, or there were face-to-face components (as part of the intervention or for assessment), i.e., to what degree got the study team to know the participant. In online-only trials, clarify if participants were quasi-anonymous and whether having multiple identities was possible or whether technical or logistical measures (e.g., cookies, email confirmation, phone calls) were used to detect/prevent these.

	1	2	3	4	5	
subitem not at all important	○	○	○	◉	○	essential

Clear selection

Does your paper address subitem 4a-ii? *

Copy and paste relevant sections from the manuscript (include quotes in quotation marks "like this" to indicate direct quotes from your manuscript), or elaborate on this item by providing additional information not in the ms, or briefly explain why the item is not applicable/relevant for your study

"Individuals who were interested in participating in the study visited the registration link where they were screened by completing a Web-based survey on basic contact information and age. The experimenter then provided eligible individuals a Zoom appointment link, where individuals indicated their preferred experiment day and time. A Zoom link was given to individuals upon their completion of booking. At the scheduled Zoom experimental session, participants were given detailed information about the study aims, length of the program, and participant involvement. Participants provided informed consent by checking the “I agree” button at the end of the study description page. Afterwards, participants received another Web-based questionnaire link to complete the pre-experiment questionnaire. Participants were then randomly assigned to one of the four experimental conditions through block randomization. Participants completed the pre-, post-, and 1-week follow-up questionnaires on the Web."

4a-iii) Information giving during recruitment

Information given during recruitment. Specify how participants were briefed for recruitment and in the informed consent procedures (e.g., publish the informed consent documentation as appendix, see also item X26), as this information may have an effect on user self-selection, user expectation and may also bias results.

	1	2	3	4	5	
subitem not at all important	○	○	○	◉	○	essential

Clear selection

Does your paper address subitem 4a-iii?

Copy and paste relevant sections from the manuscript (include quotes in quotation marks "like this" to indicate direct quotes from your manuscript), or elaborate on this item by providing additional information not in the ms, or briefly explain why the item is not applicable/relevant for your study

"At the scheduled Zoom experimental session, participants were given detailed information about the study aims, length of the program, and participant involvement. Participants provided informed consent by checking the “I agree” button at the end of the study description page."

4b) Settings and locations where the data were collected

Does your paper address CONSORT subitem 4b? *

Copy and paste relevant sections from the manuscript (include quotes in quotation marks "like this" to indicate direct quotes from your manuscript), or elaborate on this item by providing additional information not in the ms, or briefly explain why the item is not applicable/relevant for your study

"Participants completed the pre-, post-, and 1-week follow-up questionnaires on the Web."

4b-i) Report if outcomes were (self-)assessed through online questionnaires

Clearly report if outcomes were (self-)assessed through online questionnaires (as common in web-based trials) or otherwise.

	1	2	3	4	5	
subitem not at all important	○	○	●	○	○	essential

Clear selection

Does your paper address subitem 4b-i? *

Copy and paste relevant sections from the manuscript (include quotes in quotation marks "like this" to indicate direct quotes from your manuscript), or elaborate on this item by providing additional information not in the ms, or briefly explain why the item is not applicable/relevant for your study

"At the end of each Web page experience, participants were provided with a questionnaire link, of which measures microaggression, public stigma, social distance from people with mental illness, perceived autonomy, and immersiveness. One week after the experimental session, participants completed the follow-up questionnaire assessing microaggression, public stigma, and social distance from people with mental illness."

4b-ii) Report how institutional affiliations are displayed

Report how institutional affiliations are displayed to potential participants [on ehealth media], as affiliations with prestigious hospitals or universities may affect volunteer rates, use, and reactions with regards to an intervention.(Not a required item – describe only if this may bias results)

	1	2	3	4	5	
subitem not at all important	○	◉	○	○	○	essential

Clear selection

Does your paper address subitem 4b-ii?

Copy and paste relevant sections from the manuscript (include quotes in quotation marks "like this" to indicate direct quotes from your manuscript), or elaborate on this item by providing additional information not in the ms, or briefly explain why the item is not applicable/relevant for your study

"Recruitment was done through sending mass emails to students and staff at a public university in Hong Kong and by posting advertisements on social media."
This is irrelevant for the study as information related to the institution was only displayed during recruitment.

5) The interventions for each group with sufficient details to allow replication, including how and when they were actually administered

5-i) Mention names, credential, affiliations of the developers, sponsors, and owners

Mention names, credential, affiliations of the developers, sponsors, and owners [6] (if authors/evaluators are owners or developer of the software, this needs to be declared in a “Conflict of interest” section or mentioned elsewhere in the manuscript).

	1	2	3	4	5	
subitem not at all important	○	◉	○	○	○	essential

Clear selection

Does your paper address subitem 5-i?

Copy and paste relevant sections from the manuscript (include quotes in quotation marks "like this" to indicate direct quotes from your manuscript), or elaborate on this item by providing additional information not in the ms, or briefly explain why the item is not applicable/relevant for your study

No developers were involved in the study.

5-ii) Describe the history/development process

Describe the history/development process of the application and previous formative evaluations (e.g., focus groups, usability testing), as these will have an impact on adoption/use rates and help with interpreting results.

	1	2	3	4	5	
subitem not at all important	○	◉	○	○	○	essential

Clear selection

Does your paper address subitem 5-ii?

Copy and paste relevant sections from the manuscript (include quotes in quotation marks "like this" to indicate direct quotes from your manuscript), or elaborate on this item by providing additional information not in the ms, or briefly explain why the item is not applicable/relevant for your study

There was no previous attempts to evaluate the effect of the intervention. Adoption/ use rate was not applicable as it was a one-off intervention.

5-iii) Revisions and updating

Revisions and updating. Clearly mention the date and/or version number of the application/intervention (and comparator, if applicable) evaluated, or describe whether the intervention underwent major changes during the evaluation process, or whether the development and/or content was “frozen” during the trial. Describe dynamic components such as news feeds or changing content which may have an impact on the replicability of the intervention (for unexpected events see item 3b).

	1	2	3	4	5	
subitem not at all important	○	○	◉	○	○	essential

Clear selection

Does your paper address subitem 5-iii?

Copy and paste relevant sections from the manuscript (include quotes in quotation marks "like this" to indicate direct quotes from your manuscript), or elaborate on this item by providing additional information not in the ms, or briefly explain why the item is not applicable/relevant for your study

There were no revisions and updating after the trial commencement.

5-iv) Quality assurance methods

Provide information on quality assurance methods to ensure accuracy and quality of information provided [1], if applicable.

	1	2	3	4	5	
subitem not at all important	○	○	◉	○	○	essential

Clear selection

Does your paper address subitem 5-iv?

Copy and paste relevant sections from the manuscript (include quotes in quotation marks "like this" to indicate direct quotes from your manuscript), or elaborate on this item by providing additional information not in the ms, or briefly explain why the item is not applicable/relevant for your study

"The presence or absence of interactivity and the presence or absence of stigma content were manipulated in the four Web pages. All Web pages involved a story. For the COMBO and STIGMA conditions, the story was identical, which was about the journey of a person experiencing mental illness stigma. The COMBO condition utilized the Amazing Adventure Against Stigma website (https://antistigma.psy.cuhk.edu.hk/). For the INTERACT and CONTROL conditions, the story was also identical and non-stigma related, illustrating a typical day of a person."

5-v) Ensure replicability by publishing the source code, and/or providing screenshots/screen-capture video, and/or providing flowcharts of the algorithms used

Ensure replicability by publishing the source code, and/or providing screenshots/screen-capture video, and/or providing flowcharts of the algorithms used. Replicability (i.e., other researchers should in principle be able to replicate the study) is a hallmark of scientific reporting.

	1	2	3	4	5	
subitem not at all important	○	○	○	◉	○	essential

Clear selection

Does your paper address subitem 5-v?

Copy and paste relevant sections from the manuscript (include quotes in quotation marks "like this" to indicate direct quotes from your manuscript), or elaborate on this item by providing additional information not in the ms, or briefly explain why the item is not applicable/relevant for your study

Please refer to Multimedia Appendix 1.

5-vi) Digital preservation

Digital preservation: Provide the URL of the application, but as the intervention is likely to change or disappear over the course of the years; also make sure the intervention is archived (Internet Archive, webcitation.org, and/or publishing the source code or screenshots/videos alongside the article). As pages behind login screens cannot be archived, consider creating demo pages which are accessible without login.

	1	2	3	4	5	
subitem not at all important	○	◉	○	○	○	essential

Clear selection

Does your paper address subitem 5-vi?

Copy and paste relevant sections from the manuscript (include quotes in quotation marks "like this" to indicate direct quotes from your manuscript), or elaborate on this item by providing additional information not in the ms, or briefly explain why the item is not applicable/relevant for your study

The interventions used in the STIGMA, INTERACT and CONTROL conditions were archived in hard drive.

5-vii) Access

Access: Describe how participants accessed the application, in what setting/context, if they had to pay (or were paid) or not, whether they had to be a member of specific group. If known, describe how participants obtained “access to the platform and Internet” [1]. To ensure access for editors/reviewers/readers, consider to provide a “backdoor” login account or demo mode for reviewers/readers to explore the application (also important for archiving purposes, see vi).

	1	2	3	4	5	
subitem not at all important	○	○	◉	○	○	essential

Clear selection

Does your paper address subitem 5-vii? *

Copy and paste relevant sections from the manuscript (include quotes in quotation marks "like this" to indicate direct quotes from your manuscript), or elaborate on this item by providing additional information not in the ms, or briefly explain why the item is not applicable/relevant for your study

"The experimenter then provided eligible individuals a Zoom appointment link, where individuals indicated their preferred experiment day and time. A Zoom link was given to individuals upon their completion of booking."
"The four Internet-based storytelling programs were administered via the Internet with four different Web pages that were displayed in the Chinese language."
"Each Web page took approximately 20 minutes to browse through."

5-viii) Mode of delivery, features/functionalities/components of the intervention and comparator, and the theoretical framework

Describe mode of delivery, features/functionalities/components of the intervention and comparator, and the theoretical framework [6] used to design them (instructional strategy [1], behaviour change techniques, persuasive features, etc., see e.g., [7, 8] for terminology). This includes an in-depth description of the content (including where it is coming from and who developed it) [1],” whether [and how] it is tailored to individual circumstances and allows users to track their progress and receive feedback” [6]. This also includes a description of communication delivery channels and – if computer-mediated communication is a component – whether communication was synchronous or asynchronous [6]. It also includes information on presentation strategies [1], including page design principles, average amount of text on pages, presence of hyperlinks to other resources, etc. [1].

	1	2	3	4	5	
subitem not at all important	○	○	○	◉	○	essential

Clear selection

Does your paper address subitem 5-viii? *

Copy and paste relevant sections from the manuscript (include quotes in quotation marks "like this" to indicate direct quotes from your manuscript), or elaborate on this item by providing additional information not in the ms, or briefly explain why the item is not applicable/relevant for your study

"Each Web page took approximately 20 minutes to browse through."
"All Web pages involved a story. For the COMBO and STIGMA conditions, the story was identical, which was about the journey of a person experiencing mental illness stigma. The COMBO condition utilized the Amazing Adventure Against Stigma website (https://antistigma.psy.cuhk.edu.hk/). For the INTERACT and CONTROL conditions, the story was also identical and non-stigma related, illustrating a typical day of a person. Interactivity was manipulated by adding interactive elements to COMBO and INTERACT conditions, where participants could choose their actions and responses in the Web pages."
"The story content in the COMBO and INTERACT conditions was organized based on the disclosure of a person with lived experience of mental illness. The person with lived experience of mental illness accompanied participants to visualize his/her microaggressive encounters in various life domains (e.g., work, family, social circle) and the public’s misunderstanding towards mental illness with the aid of visual images in the Web page. The story also incorporated messages about the interconnection between people with or without mental illness."
"The story content in the INTERACT and CONTROL conditions formulated a typical day of a person, which began with the morning routine, having breakfast, going to work, working encounters, and ending the day."

5-ix) Describe use parameters

Describe use parameters (e.g., intended “doses” and optimal timing for use). Clarify what instructions or recommendations were given to the user, e.g., regarding timing, frequency, heaviness of use, if any, or was the intervention used ad libitum.

	1	2	3	4	5	
subitem not at all important	○	○	○	◉	○	essential

Clear selection

Does your paper address subitem 5-ix?

Copy and paste relevant sections from the manuscript (include quotes in quotation marks "like this" to indicate direct quotes from your manuscript), or elaborate on this item by providing additional information not in the ms, or briefly explain why the item is not applicable/relevant for your study

"Each Web page took approximately 20 minutes to browse through."

5-x) Clarify the level of human involvement

Clarify the level of human involvement (care providers or health professionals, also technical assistance) in the e-intervention or as co-intervention (detail number and expertise of professionals involved, if any, as well as “type of assistance offered, the timing and frequency of the support, how it is initiated, and the medium by which the assistance is delivered”. It may be necessary to distinguish between the level of human involvement required for the trial, and the level of human involvement required for a routine application outside of a RCT setting (discuss under item 21 – generalizability).

	1	2	3	4	5	
subitem not at all important	○	○	○	◉	○	essential

Clear selection

Does your paper address subitem 5-x?

Copy and paste relevant sections from the manuscript (include quotes in quotation marks "like this" to indicate direct quotes from your manuscript), or elaborate on this item by providing additional information not in the ms, or briefly explain why the item is not applicable/relevant for your study

"The experimenter then provided eligible individuals a Zoom appointment link, where individuals indicated their preferred experiment day and time. A Zoom link was given to individuals upon their completion of booking. At the scheduled Zoom experimental session, participants were given detailed information about the study aims, length of the program, and participant involvement."
"Each Web page took approximately 20 minutes to browse through." "Participants viewed the respective Web page at their own pace."

5-xi) Report any prompts/reminders used

Report any prompts/reminders used: Clarify if there were prompts (letters, emails, phone calls, SMS) to use the application, what triggered them, frequency etc. It may be necessary to distinguish between the level of prompts/reminders required for the trial, and the level of prompts/reminders for a routine application outside of a RCT setting (discuss under item 21 – generalizability).

	1	2	3	4	5	
subitem not at all important	○	◉	○	○	○	essential

Clear selection

Does your paper address subitem 5-xi? *

Copy and paste relevant sections from the manuscript (include quotes in quotation marks "like this" to indicate direct quotes from your manuscript), or elaborate on this item by providing additional information not in the ms, or briefly explain why the item is not applicable/relevant for your study

It was a one-off intervention and hence, prompts/ reminders were not applicable.

5-xii) Describe any co-interventions (incl. training/support)

Describe any co-interventions (incl. training/support): Clearly state any interventions that are provided in addition to the targeted eHealth intervention, as ehealth intervention may not be designed as stand-alone intervention. This includes training sessions and support [1]. It may be necessary to distinguish between the level of training required for the trial, and the level of training for a routine application outside of a RCT setting (discuss under item 21 – generalizability.

	1	2	3	4	5	
subitem not at all important	○	○	●	○	○	essential

Clear selection

Does your paper address subitem 5-xii? *

Copy and paste relevant sections from the manuscript (include quotes in quotation marks "like this" to indicate direct quotes from your manuscript), or elaborate on this item by providing additional information not in the ms, or briefly explain why the item is not applicable/relevant for your study

No co-interventions were involved.

6a) Completely defined pre-specified primary and secondary outcome measures, including how and when they were assessed

Does your paper address CONSORT subitem 6a? *

Copy and paste relevant sections from the manuscript (include quotes in quotation marks "like this" to indicate direct quotes from your manuscript), or elaborate on this item by providing additional information not in the ms, or briefly explain why the item is not applicable/relevant for your study

"Afterwards, participants received another Web-based questionnaire link to complete the pre-experiment questionnaire."
"At the end of each Web page experience, participants were provided with a questionnaire link, which measures microaggression, public stigma, social distance from people with mental illness, perceived autonomy, and immersiveness. One week after the experimental session, participants completed the follow-up questionnaire assessing microaggression, public stigma, and social distance from people with mental illness."
"To assess one's previous experience with mental illness, the Level of Contact Report [55] was employed, where participants indicated whether they had the experiences reported in the 12 items such as “I have watched a movie or television show in which a character depicted a person with mental illness” and “I have observed persons with a severe mental illness on a frequent basis”. Higher scores indicate higher levels of previous contact with people having mental illness."
"The 21-item Public Stigma Scale-Mental Illness-Short Version (PSSMI) [56] was used to assess mental illness public stigma and personal advocacy. Each item was rated on a 6-point Likert scale from 1 (strongly disagree) to 6 (strongly agree). Sample items included “People with mental illness are a burden to society.” (public stigma), and “I wholeheartedly fight for the rights of people with mental illness.” (personal advocacy). Reverse scoring was done for personal advocacy items. Higher scores indicate higher levels of public stigma. In this study, its Cronbach alphas were .93, .95, and .94, at baseline, post, and 1-week follow-up, respectively."
"Microaggression was measured by the 17-item Mental Illness Microaggressions Scale (MIMS-P) [57], which covers assumption of inferiority, patronization, and fear of mental illness. Each item was rated on a 4-point Likert scale from 1 (strongly disagree) to 4 (strongly agree). Sample items included “If someone I'm close to told me that they had a mental illness diagnosis, I would expect them to have trouble understanding some things” (assumption of inferiority), “If someone I'm close to told me that they had a mental illness diagnosis, I would give them advice on how to remain stable” (patronization) and “If I saw a person who I thought had a mental illness in public, I would keep my distance from them” (fear of mental illness). Higher scores indicate higher levels of microaggression. In this study, the Cronbach alphas of the MIMS-P were .78, .86, and .87 at baseline, post, and 1-week follow-up, respectively."
"The 8-item Social Distancing Scale [56] was used to measure the behavioral intention to keep a social distance from people with mental illness. Participants rated the extent to which they endorsed each item from 1 (very willing) to 6 (very unwilling) on items such as “Assuming you have children, you will let persons with mental illnesses take care of your children” and “You will work with persons with mental illnesses in the same institution”. In this study, its Cronbach alphas were .83, .88, and .86, at baseline, post, and 1-week follow-up, respectively."
"To assess perceived autonomy of the Web page experience, the 10-item Self Determination Scale (SDS) [58] was used in the post-experiment questionnaire. Each item was a pair of opposite statements, in which participants rated their level of perceived choice and self-awareness with a slider from 1 (only A feels true) to 5 (only B feels true). Sample items included item 1 “A. During this web page experience, I always feel like I choose the things I do. B. During this Web page experience, I sometimes feel that it's not really me choosing the things I do” (perceived choice) and item 2 “A. During this web page experience, my emotions

sometimes seem alien to me. B. During this web page experience, my emotions always seem to belong to me" (self-awareness)". Reverse scoring was done for perceived choice items. In this study, its Cronbach alpha was .89 at post-experiment."
"The 15-item Transportation Scale [59] was used to assess participants' immersiveness in the Web experience. It had a 4-point Likert scale from 1 (very much) to 4 (not at all) on items such as "I could picture myself in the scene of the events described in the Web page". The last four items were adapted to fit with the experimental conditions. In the COMBO and STIGMA conditions, the last four items included "While reading the Web page, I had a vivid image of the avatar representing me", "While reading the Web page, I had a vivid image of the host", "While reading the Web page, I had a vivid image of the journey" and "While reading the Web page, I had a vivid image of the dialogue". In the INTERACT and CONTROL conditions, the last four items were "While reading the Web page, I had a vivid image of the avatar representing me", "While reading the Web page, I had a vivid image of my home", "While reading the Web page, I had a vivid image of my breakfast" and "While reading the Web page, I had a vivid image of my office". Items 2, 5 and 9 were framed negatively. All the items are scored in the direction that higher scores indicate higher levels of immersiveness. In this study, its Cronbach alpha was .84 at post-experiment."

6a-i) Online questionnaires: describe if they were validated for online use and apply CHERRIES items to describe how the questionnaires were designed/deployed

If outcomes were obtained through online questionnaires, describe if they were validated for online use and apply CHERRIES items to describe how the questionnaires were designed/deployed [9].

	1	2	3	4	5	
subitem not at all important	○	○	○	◉	○	essential

Clear selection

Does your paper address subitem 6a-i?

Copy and paste relevant sections from manuscript text

"Afterwards, participants received another Web-based questionnaire link to complete the pre-experiment questionnaire."
"At the end of each Web page experience, participants were provided with a questionnaire link, of which measures microaggression, public stigma, social distance from people with mental illness, perceived autonomy, and immersiveness. One week after the experimental session, participants completed the follow-up questionnaire assessing microaggression, public stigma, and social distance from people with mental illness."

6a-ii) Describe whether and how “use” (including intensity of use/dosage) was defined/measured/monitored

Describe whether and how “use” (including intensity of use/dosage) was defined/measured/monitored (logins, logfile analysis, etc.). Use/adoption metrics are important process outcomes that should be reported in any ehealth trial.

	1	2	3	4	5	
subitem not at all important	○	◉	○	○	○	essential

Clear selection

Does your paper address subitem 6a-ii?

Copy and paste relevant sections from manuscript text

It was a one-off intervention and hence, it was not applicable.

6a-iii) Describe whether, how, and when qualitative feedback from participants was obtained

Describe whether, how, and when qualitative feedback from participants was obtained (e.g., through emails, feedback forms, interviews, focus groups).

	1	2	3	4	5	
subitem not at all important	○	◉	○	○	○	essential

Clear selection

Does your paper address subitem 6a-iii?

Copy and paste relevant sections from manuscript text

No qualitative feedback was obtained. Only quantitative outcomes were obtained.

6b) Any changes to trial outcomes after the trial commenced, with reasons

Does your paper address CONSORT subitem 6b? *

Copy and paste relevant sections from the manuscript (include quotes in quotation marks "like this" to indicate direct quotes from your manuscript), or elaborate on this item by providing additional information not in the ms, or briefly explain why the item is not applicable/relevant for your study

There were no changes after the trial commenced.

7a) How sample size was determined

NPT: When applicable, details of whether and how the clustering by care provides or centers was addressed

7a-i) Describe whether and how expected attrition was taken into account when calculating the sample size

Describe whether and how expected attrition was taken into account when calculating the sample size.

	1	2	3	4	5	
subitem not at all important	○	○	○	◉	○	essential

Clear selection

Does your paper address subitem 7a-i?

Copy and paste relevant sections from manuscript title (include quotes in quotation marks "like this" to indicate direct quotes from your manuscript), or elaborate on this item by providing additional information not in the ms, or briefly explain why the item is not applicable/relevant for your study

The procedure of the study is illustrated in Figure 1.

7b) When applicable, explanation of any interim analyses and stopping guidelines

Does your paper address CONSORT subitem 7b? *

Copy and paste relevant sections from the manuscript (include quotes in quotation marks "like this" to indicate direct quotes from your manuscript), or elaborate on this item by providing additional information not in the ms, or briefly explain why the item is not applicable/relevant for your study

It was a one-off intervention and hence, interim analyses and stopping guidelines were not applicable.

8a) Method used to generate the random allocation sequence

NPT: When applicable, how care providers were allocated to each trial group

Does your paper address CONSORT subitem 8a? *

Copy and paste relevant sections from the manuscript (include quotes in quotation marks "like this" to indicate direct quotes from your manuscript), or elaborate on this item by providing additional information not in the ms, or briefly explain why the item is not applicable/relevant for your study

"Participants were then randomly assigned to one of the four experimental conditions through block randomization."

8b) Type of randomisation; details of any restriction (such as blocking and block size)

Does your paper address CONSORT subitem 8b? *

Copy and paste relevant sections from the manuscript (include quotes in quotation marks "like this" to indicate direct quotes from your manuscript), or elaborate on this item by providing additional information not in the ms, or briefly explain why the item is not applicable/relevant for your study

"Participants were then randomly assigned to one of the four experimental conditions through block randomization."

9) Mechanism used to implement the random allocation sequence (such as sequentially numbered containers), describing any steps taken to conceal the sequence until interventions were assigned

Does your paper address CONSORT subitem 9? *

Copy and paste relevant sections from the manuscript (include quotes in quotation marks "like this" to indicate direct quotes from your manuscript), or elaborate on this item by providing additional information not in the ms, or briefly explain why the item is not applicable/relevant for your study

"Participants provided informed consent by checking the “I agree” button at the end of the study description page. Afterward, participants received another Web-based questionnaire link for pre-experiment questionnaire. Participants were then randomly assigned to one of the four experimental conditions through block randomization."
The block randomization was done by computer.

10) Who generated the random allocation sequence, who enrolled participants, and who assigned participants to interventions

Does your paper address CONSORT subitem 10? *

Copy and paste relevant sections from the manuscript (include quotes in quotation marks "like this" to indicate direct quotes from your manuscript), or elaborate on this item by providing additional information not in the ms, or briefly explain why the item is not applicable/relevant for your study

All steps were operated by the author of the manuscript.

11a) If done, who was blinded after assignment to interventions (for example, participants, care providers, those assessing outcomes) and how

NPT: Whether or not administering co-interventions were blinded to group assignment

11a-i) Specify who was blinded, and who wasn’t

Specify who was blinded, and who wasn’t. Usually, in web-based trials it is not possible to blind the participants [1, 3] (this should be clearly acknowledged), but it may be possible to blind outcome assessors, those doing data analysis or those administering co-interventions (if any).

	1	2	3	4	5	
subitem not at all important	○	○	●	○	○	essential

Clear selection

Does your paper address subitem 11a-i? *

Copy and paste relevant sections from the manuscript (include quotes in quotation marks "like this" to indicate direct quotes from your manuscript), or elaborate on this item by providing additional information not in the ms, or briefly explain why the item is not applicable/relevant for your study

Participants were blinded while the researcher was not.

11a-ii) Discuss e.g., whether participants knew which intervention was the “intervention of interest” and which one was the “comparator”

Informed consent procedures (4a-ii) can create biases and certain expectations - discuss e.g., whether participants knew which intervention was the “intervention of interest” and which one was the “comparator”.

	1	2	3	4	5	
subitem not at all important	○	○	○	◉	○	essential

Clear selection

Does your paper address subitem 11a-ii?

Copy and paste relevant sections from the manuscript (include quotes in quotation marks "like this" to indicate direct quotes from your manuscript), or elaborate on this item by providing additional information not in the ms, or briefly explain why the item is not applicable/relevant for your study

Participants were blinded while the researcher was not.

11b) If relevant, description of the similarity of interventions

(this item is usually not relevant for ehealth trials as it refers to similarity of a placebo or sham intervention to a active medication/intervention)

Does your paper address CONSORT subitem 11b? *

Copy and paste relevant sections from the manuscript (include quotes in quotation marks "like this" to indicate direct quotes from your manuscript), or elaborate on this item by providing additional information not in the ms, or briefly explain why the item is not applicable/relevant for your study

"All Web pages involved a story. For the COMBO and STIGMA conditions, the story was identical, which was about the journey of a person experiencing mental illness stigma. The COMBO condition utilized the Amazing Adventure Against Stigma website (https://antistigma.psy.cuhk.edu.hk/). For the INTERACT and CONTROL conditions, the story was also identical and non-stigma related, illustrating a typical day of a person. Interactivity was manipulated by adding interactive elements to COMBO and INTERACT conditions, where participants could choose their actions and responses in the Web pages."

12a) Statistical methods used to compare groups for primary and secondary outcomes

NPT: When applicable, details of whether and how the clustering by care providers or centers was addressed

Does your paper address CONSORT subitem 12a? *

Copy and paste relevant sections from the manuscript (include quotes in quotation marks "like this" to indicate direct quotes from your manuscript), or elaborate on this item by providing additional information not in the ms, or briefly explain why the item is not applicable/relevant for your study

"All analyses were conducted using SPSS version 27.0 (IBM Corporation) and the moderation and mediation plug-in PROCESS. Categorical chi-square and one-way ANOVA were used to examine baseline differences between experimental conditions. Repeated measures ANOVA analyses with Bonferroni adjustment and post-hoc analysis were conducted to detect significant interaction effects between condition and time to see if conditions showed significant reduction in all mental illness stigma outcomes across the three time points. Mediation analysis was conducted using PROCESS Model 4 to investigate the relationship between possible mediators, perceived autonomy, and immersiveness, with all outcomes at follow-up assessment."

12a-i) Imputation techniques to deal with attrition / missing values

Imputation techniques to deal with attrition / missing values: Not all participants will use the intervention/comparator as intended and attrition is typically high in ehealth trials. Specify how participants who did not use the application or dropped out from the trial were treated in the statistical analysis (a complete case analysis is strongly discouraged, and simple imputation techniques such as LOCF may also be problematic [4]).

	1	2	3	4	5	
subitem not at all important	○	○	○	○	○	essential

Does your paper address subitem 12a-i? *

Copy and paste relevant sections from the manuscript (include quotes in quotation marks "like this" to indicate direct quotes from your manuscript), or elaborate on this item by providing additional information not in the ms, or briefly explain why the item is not applicable/relevant for your study

"A total of 263 participants were recruited in this study and completed the experimental session, pre-experiment questionnaire and post-experiment questionnaire. All but one participants (99.62%; 262/263) completed the 1-week follow-up questionnaire. The procedure of the study is illustrated in Figure 1. Demographics and baseline characteristics of 263 participants were analysed, and data from 262 participants were analysed with repeated measures ANOVA analyses and mediation analysis."

12b) Methods for additional analyses, such as subgroup analyses and adjusted analyses

Does your paper address CONSORT subitem 12b? *

Copy and paste relevant sections from the manuscript (include quotes in quotation marks "like this" to indicate direct quotes from your manuscript), or elaborate on this item by providing additional information not in the ms, or briefly explain why the item is not applicable/relevant for your study

"All analyses were conducted using SPSS version 27.0 (IBM Corporation) and the moderation and mediation plug-in PROCESS. Categorical chi-square and one-way ANOVA were used to examine baseline differences between experimental conditions. Repeated measures ANOVA analyses with Bonferroni adjustment and post-hoc analysis were conducted to detect significant interaction effects between condition and time to see if conditions showed significant reduction in all mental illness stigma outcomes across the three time points. Mediation analysis was conducted using PROCESS Model 4 to investigate the relationship between possible mediators, perceived autonomy, and immersiveness, with all outcomes at follow-up assessment."

X26) REB/IRB Approval and Ethical Considerations [recommended as subheading under "Methods"] (not a CONSORT item)

X26-i) Comment on ethics committee approval

	1	2	3	4	5	
subitem not at all important	○	○	○	◉	○	essential

Clear selection

Does your paper address subitem X26-i?

Copy and paste relevant sections from the manuscript (include quotes in quotation marks "like this" to indicate direct quotes from your manuscript), or elaborate on this item by providing additional information not in the ms, or briefly explain why the item is not applicable/relevant for your study

"Behavioral research ethics approval was obtained from the Survey and Behavioral Research Ethics Committee of the Chinese University of Hong Kong, and the study was registered on the ClinicaTrial.gov registration (Trial Registration: ClinicalTrials.gov NCT05333848)."

x26-ii) Outline informed consent procedures

Outline informed consent procedures e.g., if consent was obtained offline or online (how? Checkbox, etc.?), and what information was provided (see 4a-ii). See [6] for some items to be included in informed consent documents.

	1	2	3	4	5	
subitem not at all important	○	○	○	◉	○	essential

Clear selection

Does your paper address subitem X26-ii?

Copy and paste relevant sections from the manuscript (include quotes in quotation marks "like this" to indicate direct quotes from your manuscript), or elaborate on this item by providing additional information not in the ms, or briefly explain why the item is not applicable/relevant for your study

"At the scheduled Zoom experimental session, participants were given detailed information about the study aims, length of the program, and participant involvement. Participants provided informed consent by checking the “I agree” button at the end of the study description page."

X26-iii) Safety and security procedures

Safety and security procedures, incl. privacy considerations, and any steps taken to reduce the likelihood or detection of harm (e.g., education and training, availability of a hotline)

	1	2	3	4	5	
subitem not at all important	○	○	○	●	○	essential

Clear selection

Does your paper address subitem X26-iii?

Copy and paste relevant sections from the manuscript (include quotes in quotation marks "like this" to indicate direct quotes from your manuscript), or elaborate on this item by providing additional information not in the ms, or briefly explain why the item is not applicable/relevant for your study

Everything was addressed in the informed consent form.

RESULTS

13a) For each group, the numbers of participants who were randomly assigned, received intended treatment, and were analysed for the primary outcome

NPT: The number of care providers or centers performing the intervention in each group and the number of patients treated by each care provider in each center

Does your paper address CONSORT subitem 13a? *

Copy and paste relevant sections from the manuscript (include quotes in quotation marks "like this" to indicate direct quotes from your manuscript), or elaborate on this item by providing additional information not in the ms, or briefly explain why the item is not applicable/relevant for your study

"A total of 263 participants were recruited in this study and completed the experimental session, pre-experiment questionnaire and post-experiment questionnaire. All but one participants (99.62%; 262/263) completed the 1-week follow-up questionnaire. The procedure of the study is illustrated in Figure 1."

13b) For each group, losses and exclusions after randomisation, together with reasons

Does your paper address CONSORT subitem 13b? (NOTE: Preferably, this is shown in a CONSORT flow diagram) *

Copy and paste relevant sections from the manuscript (include quotes in quotation marks "like this" to indicate direct quotes from your manuscript), or elaborate on this item by providing additional information not in the ms, or briefly explain why the item is not applicable/relevant for your study

"A total of 263 participants were recruited in this study and completed the experimental session, pre-experiment questionnaire and post-experiment questionnaire. All but one participants (99.62%; 262/263) completed the 1-week follow-up questionnaire. The procedure of the study is illustrated in Figure 1."

13b-i) Attrition diagram

Strongly recommended: An attrition diagram (e.g., proportion of participants still logging in or using the intervention/comparator in each group plotted over time, similar to a survival curve) or other figures or tables demonstrating usage/dose/engagement.

	1	2	3	4	5	
subitem not at all important	○	○	○	◉	○	essential

Clear selection

Does your paper address subitem 13b-i?

Copy and paste relevant sections from the manuscript or cite the figure number if applicable (include quotes in quotation marks "like this" to indicate direct quotes from your manuscript), or elaborate on this item by providing additional information not in the ms, or briefly explain why the item is not applicable/relevant for your study

"The procedure of the study is illustrated in Figure 1."

14a) Dates defining the periods of recruitment and follow-up

Does your paper address CONSORT subitem 14a? *

Copy and paste relevant sections from the manuscript (include quotes in quotation marks "like this" to indicate direct quotes from your manuscript), or elaborate on this item by providing additional information not in the ms, or briefly explain why the item is not applicable/relevant for your study

"The procedure of the study is illustrated in Figure 1."

14a-i) Indicate if critical “secular events” fell into the study period

Indicate if critical “secular events” fell into the study period, e.g., significant changes in Internet resources available or “changes in computer hardware or Internet delivery resources”

	1	2	3	4	5	
subitem not at all important	◉	○	○	○	○	essential

Clear selection

Does your paper address subitem 14a-i?

Copy and paste relevant sections from the manuscript (include quotes in quotation marks "like this" to indicate direct quotes from your manuscript), or elaborate on this item by providing additional information not in the ms, or briefly explain why the item is not applicable/relevant for your study

There were no secular events.

14b) Why the trial ended or was stopped (early)

Does your paper address CONSORT subitem 14b? *

Copy and paste relevant sections from the manuscript (include quotes in quotation marks "like this" to indicate direct quotes from your manuscript), or elaborate on this item by providing additional information not in the ms, or briefly explain why the item is not applicable/relevant for your study

The trial did not ended or stop early.

15) A table showing baseline demographic and clinical characteristics for each group

NPT: When applicable, a description of care providers (case volume, qualification, expertise, etc.) and centers (volume) in each group

Does your paper address CONSORT subitem 15? *

Copy and paste relevant sections from the manuscript (include quotes in quotation marks "like this" to indicate direct quotes from your manuscript), or elaborate on this item by providing additional information not in the ms, or briefly explain why the item is not applicable/relevant for your study

"Other detailed demographics and baseline characteristics of the participants are displayed in Table 1."

15-i) Report demographics associated with digital divide issues

In ehealth trials it is particularly important to report demographics associated with digital divide issues, such as age, education, gender, social-economic status, computer/Internet/ehealth literacy of the participants, if known.

	1	2	3	4	5	
subitem not at all important	○	○	○	○	●	essential

Clear selection

Does your paper address subitem 15-i? *

Copy and paste relevant sections from the manuscript (include quotes in quotation marks "like this" to indicate direct quotes from your manuscript), or elaborate on this item by providing additional information not in the ms, or briefly explain why the item is not applicable/relevant for your study

"Other detailed demographics and baseline characteristics of the participants are displayed in Table 1."

16) For each group, number of participants (denominator) included in each analysis and whether the analysis was by original assigned groups

16-i) Report multiple “denominators” and provide definitions

Report multiple “denominators” and provide definitions: Report N’s (and effect sizes) “across a range of study participation [and use] thresholds” [1], e.g., N exposed, N consented, N used more than x times, N used more than y weeks, N participants “used” the intervention/comparator at specific pre-defined time points of interest (in absolute and relative numbers per group). Always clearly define “use” of the intervention.

	1	2	3	4	5	
subitem not at all important	○	○	○	◉	○	essential

Clear selection

Does your paper address subitem 16-i? *

Copy and paste relevant sections from the manuscript (include quotes in quotation marks "like this" to indicate direct quotes from your manuscript), or elaborate on this item by providing additional information not in the ms, or briefly explain why the item is not applicable/relevant for your study

"The procedure of the study is illustrated in Figure 1."

16-ii) Primary analysis should be intent-to-treat

Primary analysis should be intent-to-treat, secondary analyses could include comparing only "users", with the appropriate caveats that this is no longer a randomized sample (see 18-i).

	1	2	3	4	5	
subitem not at all important	○	○	○	◉	○	essential

Clear selection

Does your paper address subitem 16-ii?

Copy and paste relevant sections from the manuscript (include quotes in quotation marks "like this" to indicate direct quotes from your manuscript), or elaborate on this item by providing additional information not in the ms, or briefly explain why the item is not applicable/relevant for your study

Public Stigma towards People with Mental Illness: "Results from repeated measures ANOVA analyses indicated a significant time x condition effect (P=.002, η2 =.04) and a post-hoc analysis was conducted."
Microaggression: "The results found a significant time x condition effect (P<.001, η2 =.06) and a post-hoc analysis was carried out."

17a) For each primary and secondary outcome, results for each group, and the estimated effect size and its precision (such as 95% confidence interval)

Does your paper address CONSORT subitem 17a? *

Copy and paste relevant sections from the manuscript (include quotes in quotation marks "like this" to indicate direct quotes from your manuscript), or elaborate on this item by providing additional information not in the ms, or briefly explain why the item is not applicable/relevant for your study

Public Stigma towards People with Mental Illness: "Results from repeated measures ANOVA analyses indicated a significant time x condition effect ($P=.002$, $\eta2 =.04$) and a post-hoc analysis was conducted. In the COMBO condition, public stigma significantly decreased from baseline to post-assessment (mean difference=0.61, 95% CI 0.49 to 0.74, $P<.001$, $\eta2 =.37$) and the decrease was maintained at 1-week follow-up (mean difference=0.53, 95% CI 0.37 to 0.69, $P<.001$, $\eta2 =.37$). In the STIGMA condition, public stigma also significantly decreased from baseline to post-assessment (mean difference=0.42, 95% CI 0.30 to 0.55, $P<.001$, $\eta2 =.22$) and the decrease was maintained at 1-week follow-up (mean difference=0.34, 95% CI 0.18 to 0.50, $P<.001$, $\eta2 =.22$). In the INTERACT condition, public stigma significantly decreased from baseline to post-assessment (mean difference=0.14, 95% CI 0.02 to 0.26, $P=.02$, $\eta2 =.03$) but the effect cannot be sustained at 1-week follow-up (mean difference=0.12, 95% CI -0.04 to 0.28, $P=.22$, $\eta2 =.03$). In the CONTROL condition, the effect was not significant from baseline to post-assessment (mean difference=0.07, 95% CI -0.06 to 0.20, $P=.56$, $\eta2 =.01$) and from baseline to 1-week follow-up (mean difference=0.09, 95% CI -0.08 to 0.26, $P=.57$, $\eta2 =.01$). In terms of mean difference values, the results indicated that the effect was strongest in COMBO, followed by STIGMA and INTERACT conditions respectively. An additional post-hoc analysis was carried out to compare COMBO with STIGMA conditions, the interaction effect between interactivity and stigma content was not significant ($P=.09$)."
Microaggression: "The results found a significant time x condition effect ($P<.001$, $\eta2 =.06$) and a post-hoc analysis was carried out. Microaggression significantly decreased from baseline to post-assessment in both COMBO (mean difference=0.34, 95% CI 0.25 to 0.42, $P<.001$, $\eta2 =.31$) and STIGMA conditions (mean difference=0.28, 95% CI 0.19 to 0.36, $P<.001$, $\eta2 =.24$). The effects were sustained and strengthened at 1-week follow-up in both conditions (COMBO: mean difference=0.39, 95% CI 0.29 to 0.49, $P<.001$, $\eta2 =.31$; STIGMA: mean difference=0.33, 95% CI 0.23 to 0.43, $P<.001$, $\eta2 =.24$). In the INTERACT condition, the effect was not significant from baseline to post-assessment (mean difference=0.03, 95% CI -0.05 to 0.12, $P=1.00$, $\eta2 =.01$) and from baseline to 1-week follow-up (mean difference=0.06, 95% CI -0.04 to 0.16, $P=0.40$, $\eta2 =.01$). In the CONTROL condition, the effect was also not significant from baseline to post-assessment (mean difference=0.03, 95% CI -0.06 to 0.12, $P=1.00$, $\eta2 =.01$) and from baseline to 1-week follow-up (mean difference=-0.04, 95% CI -0.15 to 0.07, $P=1.00$, $\eta2 =.01$). The results indicated that the effect of COMBO condition was stronger than that of STIGMA condition with regard to the mean difference values. No significant interaction effect between interactivity and stigma content was found ($P=.58$) after running the additional post-hoc analysis to compare COMBO and STIGMA conditions.
Social Distance from People with Mental Illness: "The results showed a non-significant time x condition effect ($P=.25$, $\eta2 =.02$). The additional post-hoc analysis comparing COMBO and STIGMA showed no significant interaction effect between interactivity and stigma content ($P=.46$). Details of the repeated measures ANOVA analyses are shown in Table 2."
Mediating Analysis: "To compare the mediation effect of perceived autonomy and immersiveness between conditions with public stigma and microaggression, mediation analyses were performed by putting both perceived autonomy and immersiveness into PROCESS Model 4. Table 3 showed the unstandardized and standardized factor loadings for the model. A mediation model of perceived autonomy and immersiveness between conditions with public stigma and microaggression is shown in Figure 2. Mediation analysis

for social distance was not conducted because no interaction effect was observed in social distance across conditions."
"Significant indirect effects of COMBO (b=-0.19, BCa CI [-0.36, -0.03]), STIGMA (b=-0.15, BCa CI [-0.29, -0.02]), and INTERACT (b=-0.16, BCa CI [-0.32, -0.02]) on public stigma through perceived autonomy were observed. The non-significant indirect effects of COMBO (b=-0.13, BCa CI [-0.30, 0.00]), STIGMA (b=-0.13, BCa CI [-0.30, 0.00]), and INTERACT (b=-0.07, BCa CI [-0.16, 0.00]) on public stigma through immersiveness were observed. The results showed that perceived autonomy was a significant mediator between conditions and public stigma."
"Non-significant indirect effects of COMBO (b=0.07, BCa CI [-0.03, 0.17]), STIGMA (b=0.05, BCa CI [-0.03, 0.14]), and INTERACT (b=0.06, BCa CI [-0.03, 0.15]) on microaggression through perceived autonomy were found. The indirect effects of COMBO (b=-0.13, BCa CI [-0.23, -0.06]), STIGMA (b=-0.13, BCa CI [-0.23, -0.05]), and INTERACT (b=-0.07, BCa CI [-0.13, -0.02]) on microaggression through immersiveness was significant. The results showed that immersiveness was a significant mediator between conditions and microaggression."

17a-i) Presentation of process outcomes such as metrics of use and intensity of use

In addition to primary/secondary (clinical) outcomes, the presentation of process outcomes such as metrics of use and intensity of use (dose, exposure) and their operational definitions is critical. This does not only refer to metrics of attrition (13-b) (often a binary variable), but also to more continuous exposure metrics such as "average session length". These must be accompanied by a technical description how a metric like a "session" is defined (e.g., timeout after idle time) [1] (report under item 6a).

	1	2	3	4	5	
subitem not at all important	○	○	◉	○	○	essential

Clear selection

Does your paper address subitem 17a-i?

Copy and paste relevant sections from the manuscript (include quotes in quotation marks "like this" to indicate direct quotes from your manuscript), or elaborate on this item by providing additional information not in the ms, or briefly explain why the item is not applicable/relevant for your study

It was a one-off intervention.

17b) For binary outcomes, presentation of both absolute and relative effect sizes is recommended

Does your paper address CONSORT subitem 17b? *

Copy and paste relevant sections from the manuscript (include quotes in quotation marks "like this" to indicate direct quotes from your manuscript), or elaborate on this item by providing additional information not in the ms, or briefly explain why the item is not applicable/relevant for your study

Binary outcomes were not applicable.

18) Results of any other analyses performed, including subgroup analyses and adjusted analyses, distinguishing pre-specified from exploratory

Does your paper address CONSORT subitem 18? *

Copy and paste relevant sections from the manuscript (include quotes in quotation marks "like this" to indicate direct quotes from your manuscript), or elaborate on this item by providing additional information not in the ms, or briefly explain why the item is not applicable/relevant for your study

Other analyses were not performed. All the analyses performed were reported.

18-i) Subgroup analysis of comparing only users

A subgroup analysis of comparing only users is not uncommon in ehealth trials, but if done, it must be stressed that this is a self-selected sample and no longer an unbiased sample from a randomized trial (see 16-iii).

	1	2	3	4	5	
subitem not at all important	○	○	◉	○	○	essential

Clear selection

Does your paper address subitem 18-i?

Copy and paste relevant sections from the manuscript (include quotes in quotation marks "like this" to indicate direct quotes from your manuscript), or elaborate on this item by providing additional information not in the ms, or briefly explain why the item is not applicable/relevant for your study

Subgroup analysis of comparing only users was not performed.

19) All important harms or unintended effects in each group

(for specific guidance see CONSORT for harms)

Does your paper address CONSORT subitem 19? *

Copy and paste relevant sections from the manuscript (include quotes in quotation marks "like this" to indicate direct quotes from your manuscript), or elaborate on this item by providing additional information not in the ms, or briefly explain why the item is not applicable/relevant for your study

The interventions posed no harm or unintended effects as approved by the ethics committee.

19-i) Include privacy breaches, technical problems

Include privacy breaches, technical problems. This does not only include physical "harm" to participants, but also incidents such as perceived or real privacy breaches [1], technical problems, and other unexpected/unintended incidents. "Unintended effects" also includes unintended positive effects [2].

	1	2	3	4	5	
subitem not at all important	○	○	○	●	○	essential

Clear selection

Does your paper address subitem 19-i?

Copy and paste relevant sections from the manuscript (include quotes in quotation marks "like this" to indicate direct quotes from your manuscript), or elaborate on this item by providing additional information not in the ms, or briefly explain why the item is not applicable/relevant for your study

There were no privacy breaches or technical problems.

19-ii) Include qualitative feedback from participants or observations from staff/researchers

Include qualitative feedback from participants or observations from staff/researchers, if available, on strengths and shortcomings of the application, especially if they point to unintended/unexpected effects or uses. This includes (if available) reasons for why people did or did not use the application as intended by the developers.

	1	2	3	4	5	
subitem not at all important	○	○	◉	○	○	essential

Clear selection

Does your paper address subitem 19-ii?

Copy and paste relevant sections from the manuscript (include quotes in quotation marks "like this" to indicate direct quotes from your manuscript), or elaborate on this item by providing additional information not in the ms, or briefly explain why the item is not applicable/relevant for your study

The study was a quantitative research.

DISCUSSION

22) Interpretation consistent with results, balancing benefits and harms, and considering other relevant evidence

NPT: In addition, take into account the choice of the comparator, lack of or partial blinding, and unequal expertise of care providers or centers in each group

22-i) Restate study questions and summarize the answers suggested by the data, starting with primary outcomes and process outcomes (use)

Restate study questions and summarize the answers suggested by the data, starting with primary outcomes and process outcomes (use).

	1	2	3	4	5	
subitem not at all important	○	○	○	○	◉	essential

Clear selection

Does your paper address subitem 22-i? *

Copy and paste relevant sections from the manuscript (include quotes in quotation marks "like this" to indicate direct quotes from your manuscript), or elaborate on this item by providing additional information not in the ms, or briefly explain why the item is not applicable/relevant for your study

"Results supported our hypotheses that an Internet-based storytelling program with a combination of stigma content and interactivity was able to significantly reduce public stigma, microaggression immediately post-experiment and at 1-week follow-up assessment. Contrary to our hypotheses, an Internet-based storytelling program with a combination of stigma content and interactivity could not significantly reduce social distance from people with mental illness. In other words, the storytelling program was more effective in improving individuals' stigmatizing cognitions, sense of personal advocacy [4], and microaggressions that center around their everyday conversations and encounters in daily life [57] than in enhancing individuals' willingness and intention to behaviorally interact with people with mental illness on various life domains [58]."

22-ii) Highlight unanswered new questions, suggest future research

Highlight unanswered new questions, suggest future research.

	1	2	3	4	5	
subitem not at all important	○	○	○	○	◉	essential

Clear selection

Does your paper address subitem 22-ii?

Copy and paste relevant sections from the manuscript (include quotes in quotation marks "like this" to indicate direct quotes from your manuscript), or elaborate on this item by providing additional information not in the ms, or briefly explain why the item is not applicable/relevant for your study

"Interestingly, Internet-based storytelling program with interactivity-only was also found to reduce public stigma at post-assessment although the effect could not be maintained after one week. It might support our assumption on the positive relationship between interactivity and positive affect and between positive affect and reduced prejudice [37,40]. Future studies need to examine these relationships."

20) Trial limitations, addressing sources of potential bias, imprecision, and, if relevant, multiplicity of analyses

20-i) Typical limitations in ehealth trials

Typical limitations in ehealth trials: Participants in ehealth trials are rarely blinded. Ehealth trials often look at a multiplicity of outcomes, increasing risk for a Type I error. Discuss biases due to non-use of the intervention/usability issues, biases through informed consent procedures, unexpected events.

	1	2	3	4	5	
subitem not at all important	○	○	○	○	◉	essential

Clear selection

Does your paper address subitem 20-i? *

Copy and paste relevant sections from the manuscript (include quotes in quotation marks "like this" to indicate direct quotes from your manuscript), or elaborate on this item by providing additional information not in the ms, or briefly explain why the item is not applicable/relevant for your study

"This study has some limitations that deserve attention. First, our sample mainly consisted of young university students. The findings may not be generalizable to different populations. That being said, in social marketing, segmentation of our target population is essential. The present study showed that Internet-based anti-stigma storytelling programs with interactivity may be an effective tool in reducing mental illness stigma for young, educated population in the community who are comfortable and skillful in accessing information over the Internet. Furthermore, due to the homogeneous nature of our sample, moderation analysis was not carried out in the present study. Future studies can explore possible moderators of the effect of Internet-based stigma reduction interventions, for example, gender, age, and education level...."

21) Generalisability (external validity, applicability) of the trial findings

NPT: External validity of the trial findings according to the intervention, comparators, patients, and care providers or centers involved in the trial

21-i) Generalizability to other populations

Generalizability to other populations: In particular, discuss generalizability to a general Internet population, outside of a RCT setting, and general patient population, including applicability of the study results for other organizations

	1	2	3	4	5	
subitem not at all important	○	○	○	○	◉	essential

Clear selection

Does your paper address subitem 21-i?

Copy and paste relevant sections from the manuscript (include quotes in quotation marks "like this" to indicate direct quotes from your manuscript), or elaborate on this item by providing additional information not in the ms, or briefly explain why the item is not applicable/relevant for your study

"First, our sample mainly consisted of young university students. The findings may not be generalizable to different populations."

21-ii) Discuss if there were elements in the RCT that would be different in a routine application setting

Discuss if there were elements in the RCT that would be different in a routine application setting (e.g., prompts/reminders, more human involvement, training sessions or other co-interventions) and what impact the omission of these elements could have on use, adoption, or outcomes if the intervention is applied outside of a RCT setting.

	1	2	3	4	5	
subitem not at all important	○	○	◉	○	○	essential

Clear selection

Does your paper address subitem 21-ii?

Copy and paste relevant sections from the manuscript (include quotes in quotation marks "like this" to indicate direct quotes from your manuscript), or elaborate on this item by providing additional information not in the ms, or briefly explain why the item is not applicable/relevant for your study

This was not mentioned as the major focus was on effectiveness.

OTHER INFORMATION

23) Registration number and name of trial registry

Does your paper address CONSORT subitem 23? *

Copy and paste relevant sections from the manuscript (include quotes in quotation marks "like this" to indicate direct quotes from your manuscript), or elaborate on this item by providing additional information not in the ms, or briefly explain why the item is not applicable/relevant for your study

Trial Registration: ClinicalTrials.gov NCT05333848

24) Where the full trial protocol can be accessed, if available

Does your paper address CONSORT subitem 24? *

Cite a Multimedia Appendix, other reference, or copy and paste relevant sections from the manuscript (include quotes in quotation marks "like this" to indicate direct quotes from your manuscript), or elaborate on this item by providing additional information not in the ms, or briefly explain why the item is not applicable/relevant for your study

https://www.clinicaltrials.gov/ct2/show/NCT05333848?
term=NCT05333848&draw=2&rank=1

25) Sources of funding and other support (such as supply of drugs), role of funders

Does your paper address CONSORT subitem 25? *

Copy and paste relevant sections from the manuscript (include quotes in quotation marks "like this" to indicate direct quotes from your manuscript), or elaborate on this item by providing additional information not in the ms, or briefly explain why the item is not applicable/relevant for your study

There was no other funding.

X27) Conflicts of Interest (not a CONSORT item)

X27-i) State the relation of the study team towards the system being evaluated

In addition to the usual declaration of interests (financial or otherwise), also state the relation of the study team towards the system being evaluated, i.e., state if the authors/evaluators are distinct from or identical with the developers/sponsors of the intervention.

	1	2	3	4	5	
subitem not at all important	○	○	○	●	○	essential

Clear selection

Does your paper address subitem X27-i?

Copy and paste relevant sections from the manuscript (include quotes in quotation marks "like this" to indicate direct quotes from your manuscript), or elaborate on this item by providing additional information not in the ms, or briefly explain why the item is not applicable/relevant for your study

There was no conflict of interest.

About the CONSORT EHEALTH checklist

As a result of using this checklist, did you make changes in your manuscript? *

- ○ yes, major changes
- ● yes, minor changes
- ○ no

What were the most important changes you made as a result of using this checklist?

Added more details in the title and method.

How much time did you spend on going through the checklist INCLUDING making changes in your manuscript *

Approximately seven hours.

As a result of using this checklist, do you think your manuscript has improved? *

- (x) yes
- ( ) no
- ( ) Other:

Would you like to become involved in the CONSORT EHEALTH group?

This would involve for example becoming involved in participating in a workshop and writing an "Explanation and Elaboration" document

- ( ) yes
- (x) no
- ( ) Other:

Clear selection

Any other comments or questions on CONSORT EHEALTH

It is a good tool for authors to review manuscripts but it would be better if the form allows author to save or amend after submission.

STOP - Save this form as PDF before you click submit

To generate a record that you filled in this form, we recommend to generate a PDF of this page (on a Mac, simply select "print" and then select "print as PDF") before you submit it.

When you submit your (revised) paper to JMIR, please upload the PDF as supplementary file.

Don't worry if some text in the textboxes is cut off, as we still have the complete information in our database. Thank you!

Final step: Click submit !

Click submit so we have your answers in our database!

Submit

Never submit passwords through Google Forms.

This content is neither created nor endorsed by Google. Report Abuse - Terms of Service - Privacy Policy

Google Forms
